# Targeting breast cancer metabolism with a novel inhibitor of mitochondrial ATP synthesis

**DOI:** 10.18632/oncotarget.27743

**Published:** 2020-10-27

**Authors:** Myoung Sook Kim, Ramkishore Gernapudi, Yessenia Cedeño Cedeño, Brian M. Polster, Ramon Martinez, Paul Shapiro, Santosh Kesari, Elmar Nurmemmedov, Antonino Passaniti

**Affiliations:** ^1^Department of Pathology, University of Maryland School of Medicine, Baltimore, MD, USA; ^2^Department of Biochemistry & Molecular Biology and Program in Molecular Medicine, Baltimore, MD, USA; ^3^The Marlene & Stewart Greenebaum Comprehensive Cancer Center, Baltimore, MD, USA; ^4^Department of Anesthesiology, University of Maryland School of Medicine, Baltimore, MD, USA; ^5^Department of Pharmaceutical Sciences, University of Maryland School of Pharmacy, Baltimore, MD, USA; ^6^John Wayne Cancer Institute and Pacific Neuroscience Institute at Providence Saint John’s Health Center, Santa Monica, CA, USA; ^7^Research Health Scientist, The Veteran's Health Administration Research & Development Service (VAMHCS), Baltimore, MD, USA

**Keywords:** mitochondrial ATP synthase, oxygen consumption rate, ATP synthesis, reactive oxygen species

## Abstract

Inhibitors of mitochondrial respiration and ATP synthesis may promote the selective killing of respiration-competent cancer cells that are critical for tumor progression. We previously reported that CADD522, a small molecule inhibitor of the RUNX2 transcription factor, has potential for breast cancer treatment. In the current study, we show that CADD522 inhibits mitochondrial oxidative phosphorylation by decreasing the mitochondrial oxygen consumption rate (OCR) and ATP production in human breast cancer cells in a RUNX2-independent manner. The enzyme activity of mitochondrial ATP synthase was inhibited by CADD522 treatment. Importantly, results from cellular thermal shift assays that detect drug-induced protein stabilization revealed that CADD522 interacts with both α and β subunits of the F1-ATP synthase complex. Differential scanning fluorimetry also demonstrated interaction of α subunits of the F1-ATP synthase to CADD522. These results suggest that CADD522 might target the enzymatic F1 subunits in the ATP synthase complex. CADD522 increased the levels of intracellular reactive oxygen species (ROS), which was prevented by MitoQ, a mitochondria-targeted antioxidant, suggesting that cancer cells exposed to CADD522 may elevate ROS from mitochondria. CADD522-increased mitochondrial ROS levels were enhanced by exogenously added pro-oxidants such as hydrogen peroxide or *tert*-butyl hydroperoxide. Conversely, CADD522-mediated cell growth inhibition was blocked by *N*-acetyl-l-cysteine, a general ROS scavenger. Therefore, CADD522 may exert its antitumor activity by increasing mitochondrial driven cellular ROS levels. Collectively, our data suggest *in vitro* proof-of-concept that supports inhibition of mitochondrial ATP synthase and ROS generation as contributors to the effectiveness of CADD522 in suppression of tumor growth.

## INTRODUCTION

Breast cancer (BC) is the second leading cause of cancer-related deaths among women, but early detection and improved treatment options have led to increased patient survival. Despite recent advances in treatment, however, its clinical management still remains a challenge [1]. Therefore, development of new strategies that increase the therapeutic index of chemotherapeutics and decrease the incidence of the disease are needed. Chemotherapy, radiation and hormonal therapy are useful in treating advanced BC [2], however, targeting cancer metabolism has become an attractive therapeutic option [3–6].

Alterations of mitochondrial function are associated with development of several types of cancer [7]. In contrast to Warburg’s first observations, maintaining functional mitochondria appears to be key for cancer cell survival and proliferation [8]. Mitochondria are still functional in glycolytic cancer cells (with high Glut-1 and phospho-Akt expression) producing abundant amounts of ATP [9]. Recent reports suggest that inhibitors of mitochondrial oxidative phosphorylation (OXPHOS) could promote the selective eradication of stem-like tumor cells due to their preferential sensitivity to mitochondrial reactive oxygen species (ROS) [10]. Functional mitochondria that are respiration competent are thus critical for tumorigenesis and targeting mitochondrial metabolism may be a novel and successful therapeutic strategy for cancer [7].

Under physiological conditions, mitochondrial membrane proton pumps (*i.e.,* electron transport chain, ETC) generate an electrochemical proton gradient, the main component of which is mitochondrial membrane potential (MMP, ΔΨm). ATP synthase acts as a sensor of glucose supply by utilizing a proton gradient generated by the ETC from electron donors that ultimately originate from glucose-derived pyruvate. The protonmotive force (Δp) is used by ATP synthase to produce ATP to meet the energy needs of the cell. Reactive oxygen species (ROS) are primarily generated from complex I and III in mitochondria, but increased ROS is often due to blockade of complex IV [11, 12] or ATP synthase [13–15]. ATP synthase inhibition results in ΔΨm elevation, leading to increased electron leak to superoxide [15]. Mitochondrial ROS are important for cell proliferation and tumor growth [16], but also can induce DNA damage, protein oxidation and lipid peroxidation [15, 17], potentially initiating cell death [18, 19]. Intrinsic MMP (ΔΨm) in cancer cells generally correlates with tumor development and progression, and invasive cellular behavior [20].

Numerous molecules that act on mitochondria are currently used or being tested in clinical trials [21], and many therapeutics that target mitochondria reduce ATP levels and increase ROS production [15, 22–28]. For example, tamoxifen is widely used in adjuvant therapy for all stages of BC. It inhibits complexes III and IV, inducing MMP (ΔΨm) collapse [29] and increased ROS production [30]. In addition, it has been reported that the mitochondrial Fo/F1-ATP synthase is a target for dietary phytochemicals such as resveratrol, genistein, and epigallocatechin [14], which can reduce ATP levels. Resveratrol targets Complexes I and II, but it also targets the F1 domain of ATP synthase, resulting in a non-competitive inhibition of F1-ATP synthase activity [31]. Therefore, inhibiting mitochondria may be a rational therapeutic approach, since BC cells are especially sensitive to ROS-mediated oxidative stress [23].

ATP synthase is reported to be upregulated in breast tumors. Among five subunits in the hydrophilic F1-portion of the mitochondrial H^+^-ATP synthase, the α subunits are correlated with larger, poorly differentiated and high stage tumors [32, 33]. However, one report suggests no significant difference in the expression levels of β-F1-ATP synthase in BC tissues when compared with normal breast [34]. Nonetheless, reduced expression of the catalytic β subunit (β-F1-ATP synthase) is linked to cancer progression [35–37] and resistance of cancer cells to standard anticancer therapies [38–41]. β-F1-ATP synthase levels are also inversely correlated with aerobic glycolysis in cancer cells [42].

Most ATP synthase inhibitors often demonstrate unacceptable *in vivo* toxicity [43]. However, some of them still have potential to be used as anticancer agents. For example, oligomycin A inhibits the proton-translocating Fo-portion of the ATP synthase, and also affects the F1-portion at high concentration [44, 45]. Oligomycin dramatically attenuates BC metastatic seeding in the lungs, which demonstrates the functional importance of OXPHOS in metastasis and highlights its potential as a therapeutic target to prevent metastatic spread in patients with BC [46]. Aurovertin B inhibits the activity of ATP synthase by interacting with the β subunit of the F1-ATP synthase and limits proliferation of BC cells with little influence on the normal mammary epithelial cells (MCF-10A) [32]. Citreoviridin, which is in the same class as aurovertin, targets the β subunit of the F1-ATP synthase [47, 48], inhibits the growth and proliferation of BC cells as well as lung adenocarcinoma cells [49, 50]. Rhodamine123 inhibits BC colony formation, which was reported to be due to inhibition of the FoF1-ATP synthase enzyme complex leading to ATP depletion [51]. Benzodiazepine (Bz-423) directly inhibits the F1-ATP synthase and initiates apoptosis by increasing generation of superoxide (O_2_^–^) from the respiratory chain within mitochondria [43].

We previously reported that the transcription factor runt-related transcription factor 2 (RUNX2) promotes glycolytic switching in BC cells by increasing expression of genes regulating glycolytic pathways [52]. RUNX2 decreases pyruvate dehydrogenase (PDH) activity but RUNX2 KD increases mitochondrial oxygen consumption rate (OCR) by increasing the activity of PDH, a rate limiting step for entry into the TCA cycle at the branch point for pyruvate utilization. These findings led us to hypothesize that targeting RUNX2 might inhibit BC growth and/or progression by reversing tumor cell dependence on glycolysis. In our effort to find small molecules that target RUNX2 by interfering with RUNX2 binding to the specific DNA sequences, we used Computer-Assisted Drug Design (CADD) [52, 53]. Based on this approach, CADD522 (Supplementary Figure 2, left) was identified as a potent inhibitor of RUNX2-DNA binding [52, 53].

CADD522 inhibited the *in vitro* DNA binding activity of RUNX2 at nanomolar concentrations (IC50 ≅ 10 nM) [53] and inhibited BC growth and metastasis in both *in vitro* and *in vivo* models by specifically downregulating RUNX2-mediated transcription of downstream targets [52]. Since CADD522 suppressed RUNX2-mediated Glut-1 expression as well as glucose uptake and utilization [52], we initially hypothesized that CADD522 could restore the mitochondrial metabolic pathways that RUNX2 repressed. Unexpectedly, however, we observed that CADD522 directly targeted mitochondrial oxidative phosphorylation, decreasing mitochondrial OCR and ATP production in BC cells. CADD522 repressed FoF1-ATP synthase activity but increased ROS levels in a RUNX2-independent manner. Therefore, CADD522 may demonstrate therapeutic potential for BC by targeting mitochondria.

## RESULTS

### Inhibition of mitochondrial respiration

Mitochondrial respiration is the most important generator of cellular energy through the process of energy conversion of carbon substrates into ATP. Previously, we showed that loss of RUNX2 by gene knockdown (KD) increases OCR [54]. Our extended studies now demonstrate that ATP levels and mitochondrial ATP synthase activity significantly increase in Hs578t cells after RUNX2 KD compared to non-targeting control (NTC) (Supplementary Figure 1A and 1B). The increase was observed in Galactose M, a condition where cellular ATP production is dependent on mitochondrial OXPHOS rather than glycolysis, but not in Serum-Free Glucose M. Mitochondrial Complex I activity also increased in RUNX2 KD cells compared to NTC cells (Supplementary Figure 1C). In contrast to a slight but significant increase of mitochondrial mass in RUNX2 KD cells (Supplementary Figure 1D), no significant difference in MMP (ΔΨm) was observed between NTC and RUNX2 KD cells (Supplementary Figure 1E). In addition, intracellular and mitochondrial ROS levels determined by DCF and MitoSox fluorescence intensity, respectively, as well as the H_2_O_2_ Luc intensity increased in Hs578t cells with RUNX2 KD (Supplementary Figure 1F and 1G). Collectively, these findings indicate that RUNX2 KD results in activation of mitochondrial ATP synthase activity, which may lead to increases in respiration [54], ATP production and ROS generation.

**Figure 1:**
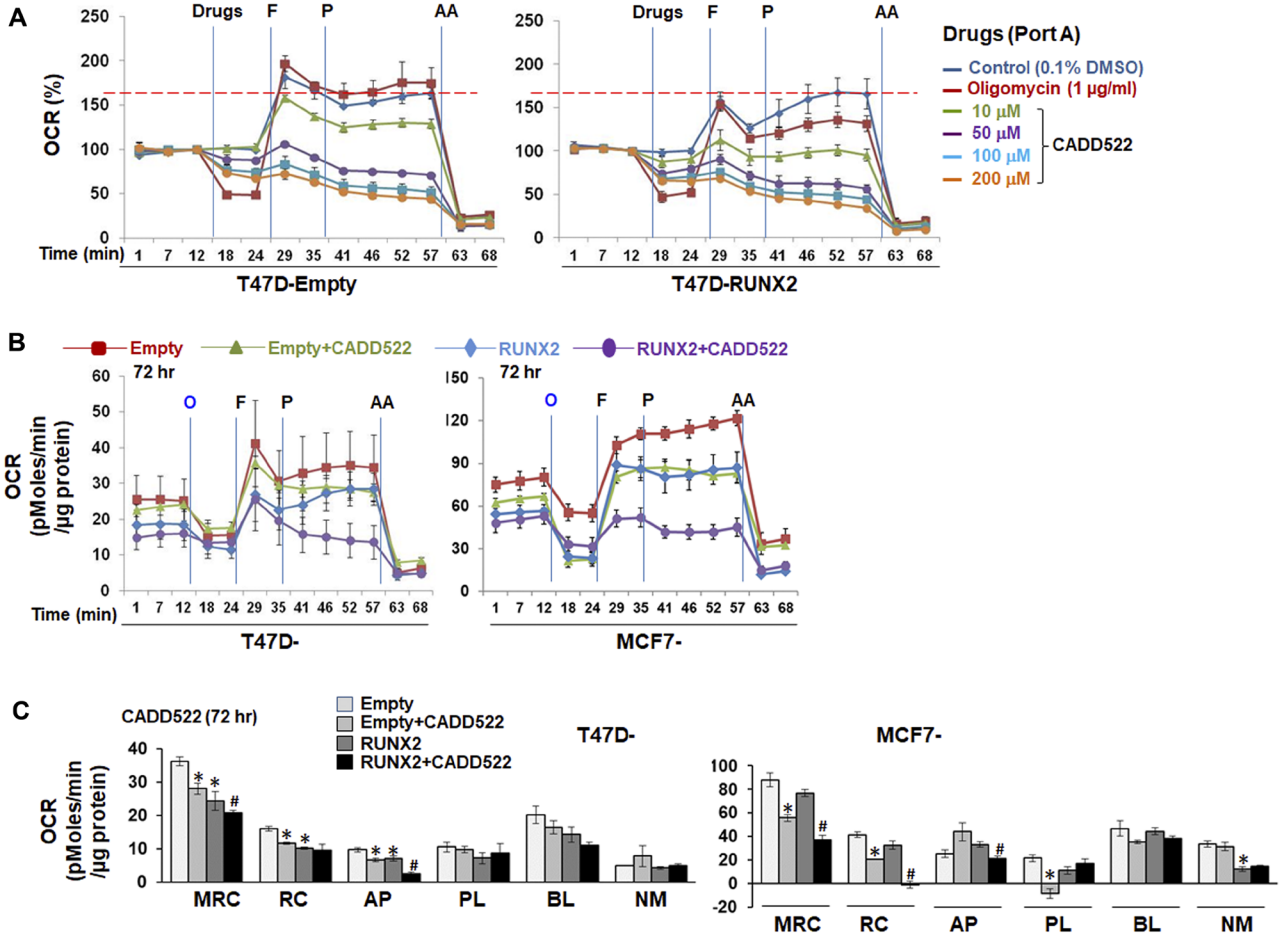
Inhibitory effect of CADD522 on mitochondrial OCR. (**A**) Acute OCR changes were determined in T47D-RUNX2 and -Empty cells with no prior CADD522 treatment. Cells seeded in Seahorse plates were incubated overnight in growth medium without G418, and then replenished with Seahorse-Certified Medium on the assay day. Vehicle control (0.1% DMSO), Oligomycin or CADD522 were directly injected into port A of the XF Extracellular Flux Analyzer, and OCR was measured. Data (% OCR) are presented as the percentage of the basal OCR value at t = 12 min (100%). Experiments were performed in four replicates and repeated twice (mean ± SD). O, Oligomycin (1 μg/ml); F, FCCP (1 μM, port B); P, pyruvate (10 mM, port C); AA, antimycin A (1 μM, port D). Red line, maximal OCR value of the Control at t = 57 min. (**B**) Cells were treated with CADD522 for 6~72 hr and replenished with Seahorse-Certified Medium with no CADD522. OCR was measured in the XF Extracellular Flux Analyzer. Data (mean ± SD) are presented as OCR (pMoles/min/μg protein). (**C**) Mitochondrial respiratory parameters were calculated as described in Material and Methods: Maximal respiratory capacity (MRC), mitochondrial reserve capacity (RC), ATP production-linked respiration (AP), proton leak-linked respiration (PL), baseline respiration (BL), and non-mitochondrial respiration (NM). Experiments were performed in four replicates and data are expressed as mean ± SE from two independent experiments. ^*^ *P < 0.05* compared to Empty cells with vehicle control (0.1% DMSO); ^#^ *P < 0.05* compared to RUNX2-expressing cells with vehicle control.

These findings led us to hypothesize that CADD522 (Supplementary Figure 2A, left), a RUNX2-DNA binding inhibitor, would promote mitochondrial OCR and ATP synthesis in BC cells expressing RUNX2. To test this hypothesis in this study, we first determined the kinetics of the acute (immediate) OCR changes in T47D-Empty and -RUNX2 overexpressing cells by exposing cells to CADD522 at the time of assay. Increasing concentrations of CADD522 (0~200 μM) were directly injected into port A of the XF analyzer. In parallel, oligomycin A was injected in separate wells as a positive control. Unexpectedly, CADD522 reduced the respiratory capacity of both T47D-Empty and T47D-RUNX2 cells in a concentration-dependent manner (Figure 1A). Oligomycin A had no significant influence on the FCCP-induced maximal respiratory capacity (MRC) compared to the vehicle control (Control, 0.1% DMSO).

Next, we treated cells with CADD522 (50 μM) for 72 hr and determined the OCR without further addition of CADD522 during the assay. Mitochondrial respiration parameters-baseline OCR (BL), maximal respiratory capacity (MRC), reserve capacity (RC), ATP production-linked OCR (AP), and proton leak-linked OCR (PL) (Supplementary Figure 2A, right) were measured by the addition of mitochondrial OXPHOS inhibitors such as oligomycin A (O) or antimycin A (AA) [55]. Among the mitochondrial respiration parameters, CADD522-mediated inhibition was mostly observed in the MRC, RC and AP (Figure 1B and 1C). The percent (%) inhibition of MRC and RC was more profound in MCF7-RUNX2 cells than MCF7-Empty cells, which was also in a time-dependent manner (Supplementary Figure 2B). Similar results were observed in cells treated with CADD522 for less than 30 hr (Supplementary Figure 2C and 2D). These results indicate that CADD522 significantly inhibits OCR in both ectopic RUNX2-expressing T47D and MCF7 cells as well as their Empty controls. Mean values of OCR and % inhibition of individual parameters are summarized in Supplementary Table 1.

**Figure 2:**
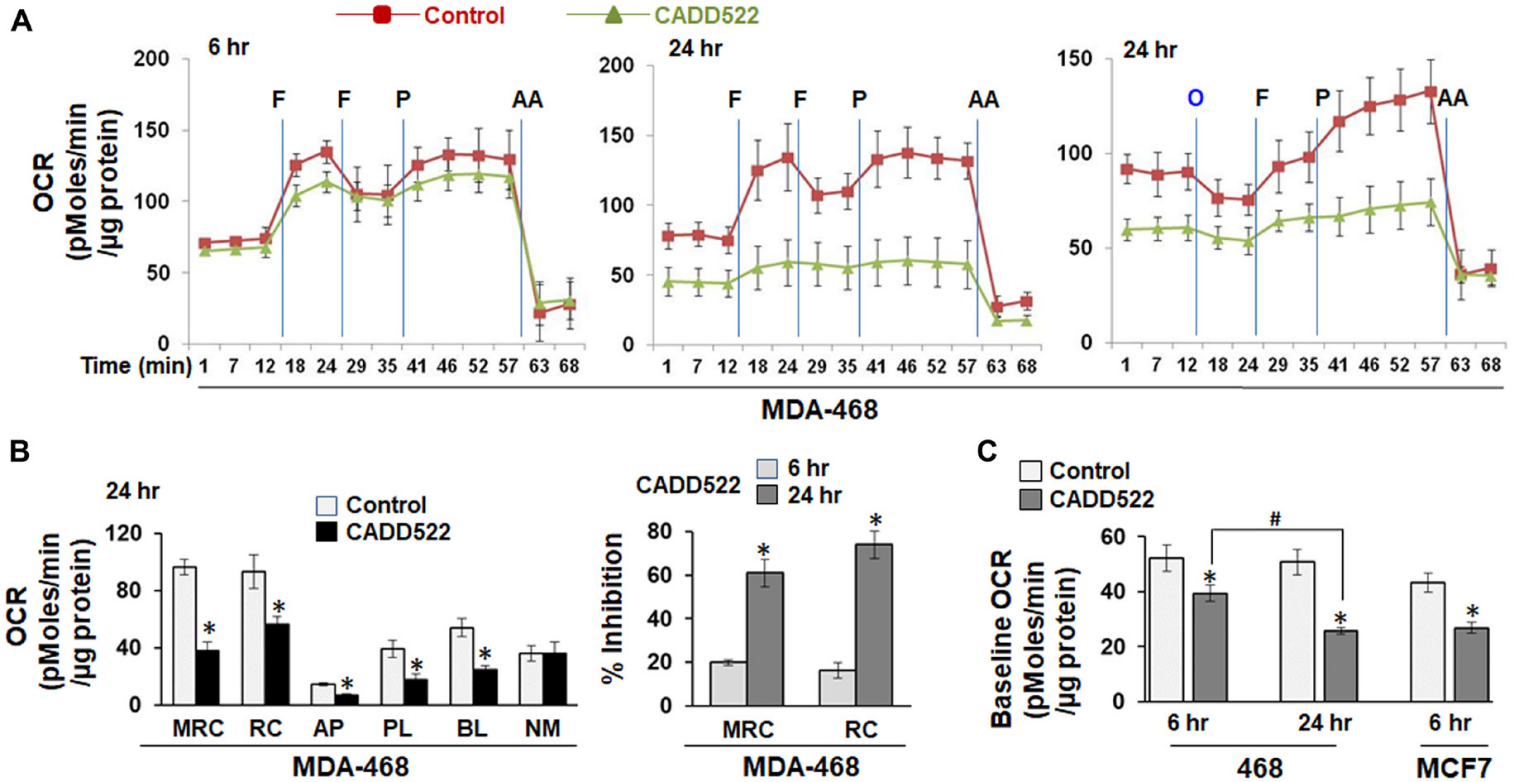
Inhibition of mitochondrial OCR in CADD522-treated BC cells. (**A**) MDA-468 cells were treated with CADD522 for 6 and 24 hr and replenished with Seahorse-Certified Medium with no CADD522. Data (mean ± SD) are presented as OCR (pMoles/min/μg protein). O, Oligomycin (1 μg/ml); F, FCCP (1 μM, port B); P, pyruvate (10 mM, port C); AA, Antimycin A (1 μM, port D). (**B**) Mitochondrial respiration parameters in MDA-468 cells treated with CADD522 for 24 hr (left). Experiments were performed in four replicates and data are presented as mean ± SE from two independent experiments. ^*^ *P < 0.05* compared to Control. Percent (%) inhibition of MRC and RC in MDA-468 cells treated with CADD522 (right). ^*^ *P < 0.05* compared to % inhibition at 6 hr of treatment. (**C**) Baseline cellular OCR (BL) was calculated from basal respiration (value at t = 12 min) after subtracting NM. Experiments were done in four replicates and repeated twice (mean ± SE). ^*^ *P < 0.05* compared to Control; ^#^ *P < 0.05* compared to BL of CADD522-treated cells for 6 hr.

To exclude the possibility that long-term use of G418 for selection and maintenance of stable cell lines might affect cellular OCR response to CADD522, we determined OCR in MDA-468 and MCF7 cells after exposure to CADD522 in the absence of G418. The OCR was determined in the absence of CADD522 at the time of analysis. CADD522 treatment for 6 hr in MDA-468 cells (Figure 2A) or MCF7 cells (Supplementary Figure 3A) did not have dramatic effects on the OCR compared to the vehicle control, whereas 24 hr of CADD522 treatment significantly suppressed the mitochondrial respiration parameters in MDA-468 cells (Figure 2A–2C). The percent (%) Inhibition of MRC and RC increased in a time-dependent manner (Figure 2B, right).

In addition, CADD522 significantly suppressed the baseline OCR (BL) of MDA-468 and MCF7 cells (Figure 2C). In MDA-468 cells, CADD522-mediated inhibition was greater after 24 hr than after 6 hr despite no difference between vehicle controls. Inhibition of the baseline OCR was clearly observed in both T47D-Empty and -RUNX2 cells treated with CADD522 for 72 hr, as well as in MCF7-Empty and -RUNX2 cells with CADD522 for 30 hr (Supplementary Figure 3B). However, no significant difference was observed in the non-mitochondrial (antimycin A-resistant) OCR (NM, value at t = 63 min) between vehicle- and CADD522-treated cells (Supplementary Figure 3C and Figure 1C), indicating that CADD522 targets mitochondria rather than other cellular sources of respiration. Taken together, our data clearly show that CADD522 represses the key mitochondrial respiration parameters. Our previous results showed that among thirteen BC cells lines tested, MDA-468 and MCF7 cells displayed the highest sensitivity to CADD522 in short- and long-term cell growth assays even though MDA-468 and MCF7 cells express low levels of RUNX2 [52]. Therefore, CADD522 may target primarily mitochondria in BC cells with low levels of RUNX2.

### Decrease in mitochondrial ATP production

**Figure 3:**
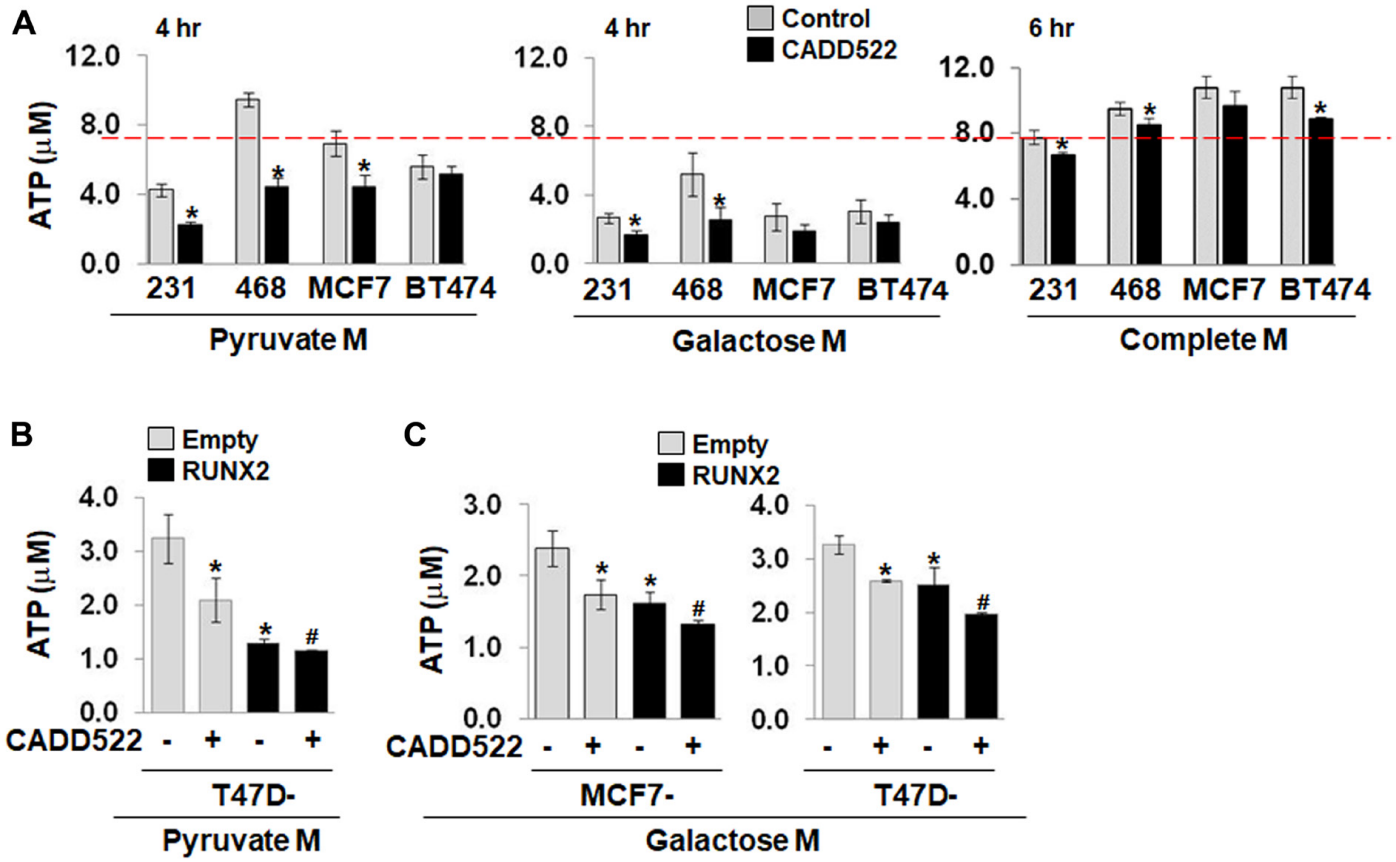
CADD522 treatment suppresses the levels of ATP. (**A**) Cells were treated with CADD522 (50 μM) in Pyruvate M (4 hr), Galactose M (4 hr) or Complete M (6 hr), and cellular ATP levels were determined. Galactose M, DMEM with 5 mM galactose, 1 mM pyruvate, no glucose, 2 mM glutamine, 10% serum; Pyruvate M, DMEM with 2 mM pyruvate, no Glucose, no Glutamine with 5% serum. Complete M, 25 mM glucose, 2 mM glutamine, 1 mM pyruvate, 10% serum. Experiments were done in four replicates and repeated twice (mean ± SD). ^*^ *P < 0.05* compared to vehicle control (0.1% DMSO). Red line, ATP level of the control MDA-231 cells in Complete M. (**B**, **C**) Ectopic RUNX2 expressing cells and their Empty controls were treated with CADD522 in Pyruvate M (4 hr) or Galactose M (6 hr), and cellular ATP levels were determined. ^*^ *P < 0.05* compared to the vehicle control of Empty cells; ^#^ *P < 0.05* compared to the vehicle control of ectopic RUNX2-expressing cells.

When cells have limited glucose availability, ATP is synthesized mainly from mitochondria. Cells in culture medium with pyruvate or galactose but not glucose are primarily dependent on OXPHOS as glycolysis contribution to ATP generation is minimized. In contrast, under serum-free, glucose-supplemented conditions (Serum-Free Glucose M), normal mitochondrial function is compromised, and mitochondrial ATP production is suppressed. Indeed, we observed that BC cells in Pyruvate M or Galactose M exhibited lower levels of ATP than in Complete M owing to suppression of ATP production derived from glycolysis and possible changes in energy demand (Figure 3A, Supplementary Figure 4A).

To examine whether CADD522-induced OCR changes have a functional effect on ATP levels, we treated cells with CADD522 for a short period of time (4~6 hr) and determined changes in ATP levels by bioluminescence assay. CADD522 significantly inhibited ATP levels in BC cells, which was greater in cells incubated in media without glucose (Pyruvate M, Galactose M) than in complete M (Figure 3A). Consistently, ATP levels decreased in both ectopic RUNX2-expressing MCF7 and T47D cells and their Empty controls when glycolytic ATP generation was blocked (Figure 3B and 3C). However, CADD522 had little effect on ATP levels when cells were incubated in Serum-Free Glucose M (Supplementary Figure 4B), indicating that these cells are sensitive to CADD522-mediated ATP inhibition primarily under conditions when they depend on mitochondrial OXPHOS. Decreased ATP levels in response to CADD522 were observed when cells were treated with CADD522 for a relatively long period of time (24~48 hr) (Supplementary Figure 4C), and also were generally observed in BC cells regardless of their RUNX2 status.

### Increased mitochondrial ROS generation

**Figure 4:**
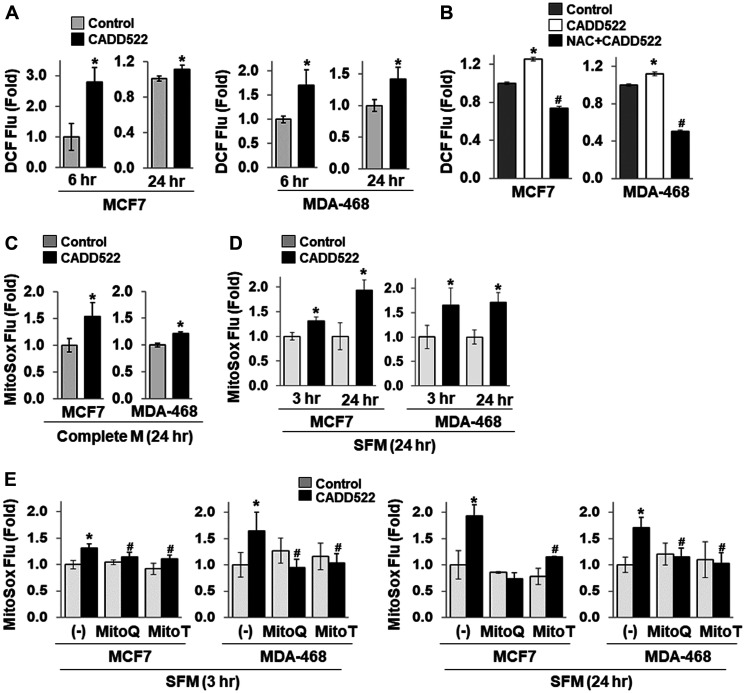
CADD522 increases ROS level. (**A**, **B**) Cells were treated with CADD522 (50 μM) for 6 or 24 hr in complete growth medium, and intracellular ROS levels were determined with fluorescence-based CM-H_2_DCFDA. pH-adjusted NAC (5 mM, pH 7.2) was pre-treated for 1 hr and further incubated with CADD522 for 24 hr. Data (DCF fluorescence relative to Calcein green fluorescence) are presented as Fold, which was calculated from the relative value to the average of the vehicle control (0.1% DMSO, = 1). All determinations were in four replicates and repeated twice (mean ± SD). ^*^ *P < 0.05* compared to the control; ^#^ *P < 0.05* compared to CADD522 alone. (**C**) Cells were treated with CADD522 for 24 hr in complete growth medium, and mitochondrial superoxide levels were measured by incubating cells with MitoSox Red dye. (**D**) Cells were treated with CADD522 for 3 or 24 hr in serum-free medium (SFM), and mitochondrial superoxide levels were determined. (**E**) Mitochondria-specific antioxidants MitoQ (5 μM) and MitoTempo (MitoT, 50 μM) were pre-treated for 1 hr and further incubated with CADD522 for 24 hr in serum-free condition.

Mitochondria are a source as well as a target of ROS. Leakage of electrons from the ETC produces superoxide radicals. Mitochondrial respiration normally restricts ROS production since electron flow is very efficient, but suppression of respiration and ATP production result in a greatly enhanced rate of free radical production [15]. ROS consist of the oxygen radical superoxide anion (O_2_^•−^) and hydroxyl radical (•OH) and non-radical oxygen species, such as hydrogen peroxide (H_2_O_2_) and singlet oxygen (O_2_). To determine whether inhibition of respiration (OCR) by CADD522 increases intracellular ROS levels, we treated cells with CADD522 for 6 or 24 hr and incubated cells with the fluorogenic dye CM-H_2_DCFDA that measures hydroxyl, peroxyl, and other ROS within cells [56]. CADD522 significantly increased intracellular ROS production in both MCF7 and MDA-468 cells (Figure 4A). However, co-treatment with *N*-acetyl-l-cysteine (NAC, 5 mM), a widely used antioxidant [57, 58], diminished the CADD522-increased intracellular ROS levels to below the level of the control (Figure 4B). We further determined mitochondrial ROS levels in cells treated with CADD522 for 24 hr using MitoSox Red, a fluorescent dye relatively specific for superoxide. As shown in Figure 4C and 4D, CADD522 increased the level of mitochondrial ROS in MCF7 and MDA-468 cells, which was more evident in serum-free than serum-containing condition. Similar results were observed in CADD522-treated ectopic RUNX2-expressing T47D and MCF7 cells as well as their Empty controls (Supplementary Figure 5A and 5B). In addition, CADD522-regulated mitochondrial ROS levels were significantly reduced by mitochondria-targeted antioxidants MitoQ (5 μM) [59] and MitoTEMPO (MitoT, 50 μM) [60] (Figure 4E). These results indicate that part of ROS levels increased by CADD522 may be derived from mitochondria.

### Inhibition of ATP synthase enzymatic activity

**Figure 5:**
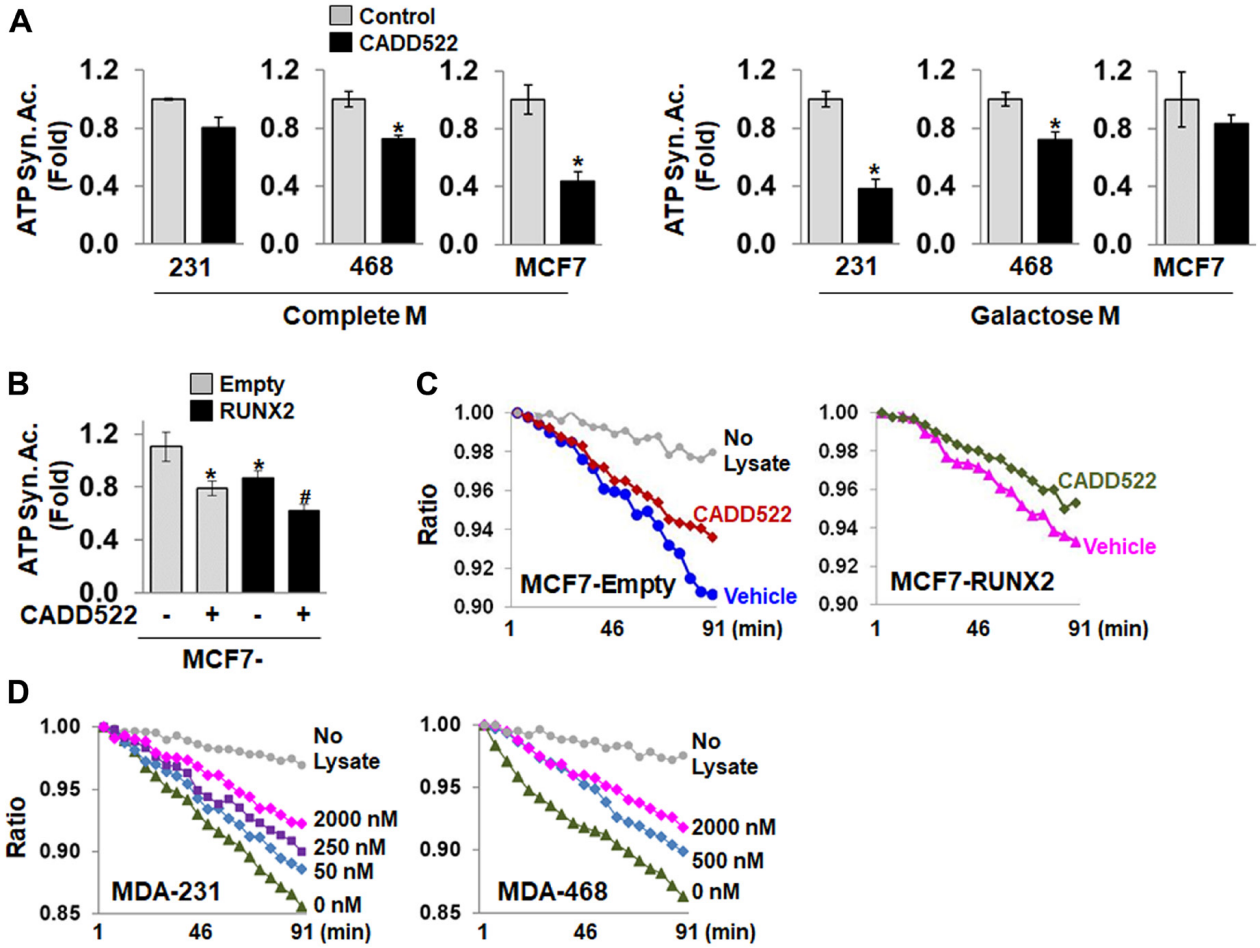
CADD522 inhibits mitochondrial ATP synthase activity. (**A**) Cells were treated with CADD522 for 24 hr in Complete M or 4 hr in Galactose M, and ATP synthase activity (ATP Syn. Ac.) was determined in cell lysates (50 μl) using commercial kits according to the manufacturer’s instruction. Absorbance was measured every 2 min (kinetic program) at 340 nm. Fold-differences were calculated from the relative activity to the average of the vehicle control (0.1% DMSO, = 1). All determinations were in triplicate and repeated twice (mean ± SD). ^*^ *P < 0.05* compared to Control. (**B**) MCF7-RUNX2 and MCF7-Empty cells were treated with CADD522 for 24 hr in Complete M, and mitochondrial ATP synthase activity was determined. ^*^ *P < 0.05* compared to MCF7-Empty cells with vehicle treatment. Experiments were performed in triplicate and repeated twice (mean ± SD). ^#^ *P < 0.05* compared to MCF7-RUNX2 with vehicle treatment. (**C**) A representative result of mitochondrial ATP synthase activity showing changes of Ratio over time. Ratio, relative absorbance to the absorbance at 0 min. (**D**) Cell lysates (50 μg) isolated from MDA-231 and MDA-468 cells that were not treated with CADD522 were directly incubated with CADD522 for 30 min, and the *in vitro* ATP synthase activity was determined. 0.05% DMSO was used as vehicle control. Relative activity is expressed as changes in absorbance or Ratio per minute (ΔAbsorbance or ΔRatio)/Δmin.

OCR is regulated by both ETC complex activity and the rate of ATP production from mitochondrial H^+^-ATP synthase, a nuclear genome-encoded enzyme that is a central player in defining the bioenergetic activity of the cell. To investigate how CADD522 suppresses the level of ATP, we incubated BC cells in Complete M with CADD522 (50 μM) for 24 hr, and then isolated cell lysates to perform quantitative measurement of the mitochondrial ATP synthase activity. The ATP synthase enzyme was immunocaptured within the wells of a microplate and CADD522 was not further added during the assay. CADD522 significantly inhibited the activity of ATP synthase in BC cells (Figure 5A, left). Significant decrease of ATP synthase activity was also observed in ectopic RUNX2-expressing MCF7 cells treated with CADD522 for 24 hr (Figure 5B and 5C, Supplementary Figure 6A). Similar inhibition was observed even after a short treatment time in Galactose M (4 hr) (Figure 5A, right). These results indicate that reduced ATP synthase activity persists after CADD522 was removed during isolation of protein lysates.

To rule out that the inhibition could have been due to blocking upstream components of the ETC, protein lysates were prepared from MDA-231 and MDA-468 cells without prior CADD522 treatment. After immunocapture with specific antibody, the ATP synthase enzyme was incubated with CADD522 (0~2 μM) *in vitro* for 30 min. As seen in Figure 5D and Supplementary Figure 6C, CADD522 decreased the slope of ATP synthase (*i.e.*, inhibition of the activity) in either MDA-231 or MDA-468 cells in a concentration-dependent manner. These results suggest that lower ATP levels in response to CADD522 could be due to direct inhibition of the mitochondrial ATP synthase activity. The mean values of the ATP synthase activity of individual cells are shown in Supplementary Table 2.

### No changes in MMP or mitochondrial Complex I activity

Under physiological conditions, mitochondrial membrane proton pumps of the ETC generate an electrochemical proton gradient, the main component of which is MMP (ΔΨm). Proton pumping promotes normal respiratory function and synthesis of ATP to meet the energy needs of the cell. To determine the effect of CADD522 on MMP (ΔΨm), we incubated BC cells with a fluorescent ΔΨm-sensitive dye, JC-1. However, we did not observe any significant difference of JC-1 fluorescence intensity between cells treated with vehicle and CADD522 for 4~72 hr (Supplementary Figure 7A). Changes of JC-1 fluorescence by CADD522 were also not observed in cells incubated with TMRM, another ΔΨm-detecting dye. These results show that CADD522 targets mitochondria without causing significant collapse of ΔΨm.

Complex I (NADH dehydrogenase) is the primary electron acceptor from the TCA cycle metabolite NADH. A blockade of the terminal OXPHOS step (mitochondrial ATP synthase) can also feedback to slow complex I activity, preventing efficient transfer of electrons, and thus leading to increased ROS [61]. We determined complex I activity under the same cell conditions described for measuring mitochondrial ATP synthase activity. However, complex I activity was not altered by CADD522 treatment for up to 72 hr in all BC cells examined (Supplementary Figure 7B).

### Interaction of α and β subunits of F1-ATP synthase with CADD522

**Figure 6:**
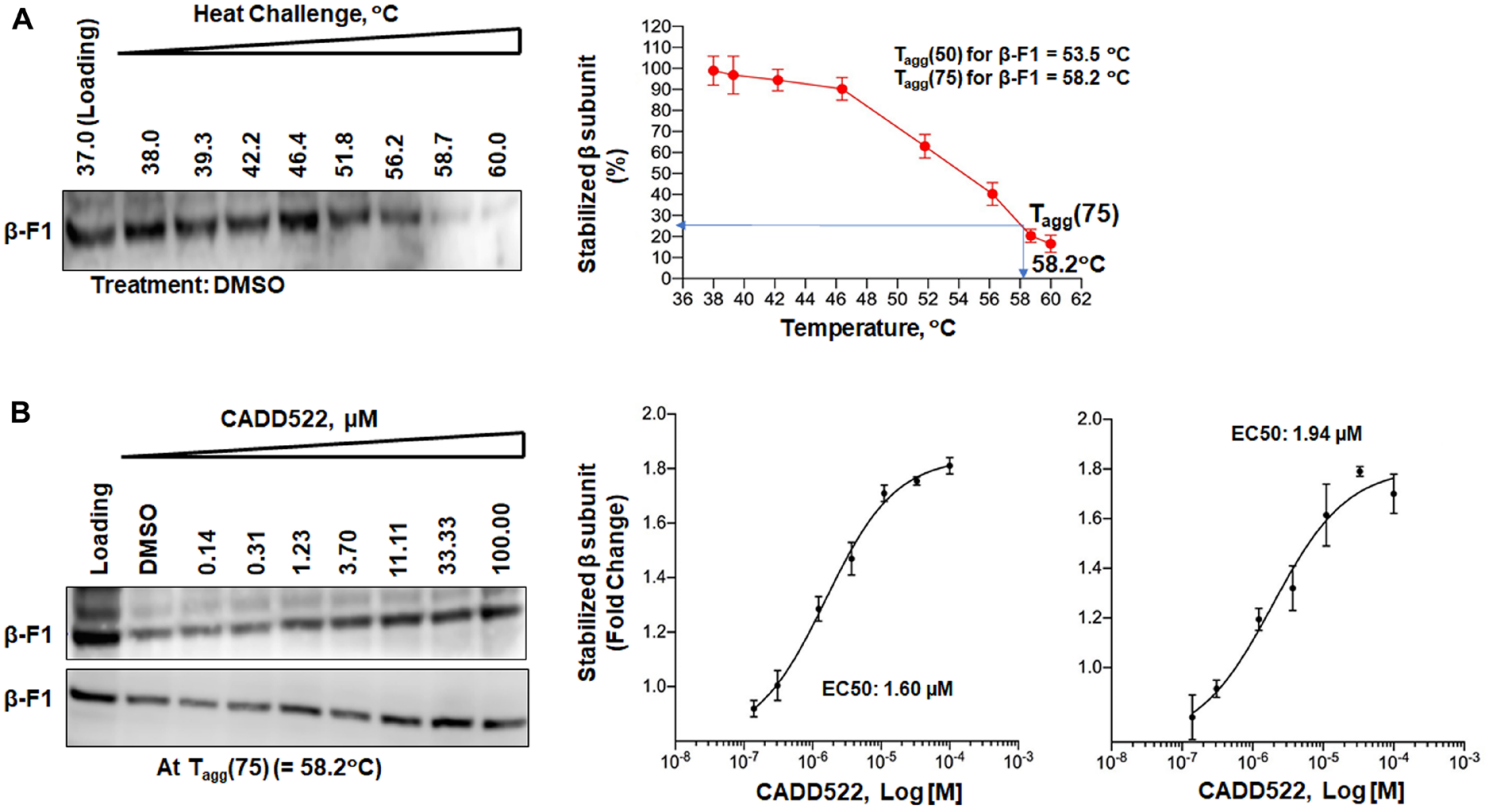
Engagement of CADD522 with the mitochondrial F1-ATP synthase, β subunit. (**A**) Left: representative immunoblot result of the β-F1-ATP synthase upon heat challenges. Multiple aliquots of cell lysate isolated from MDA-231 cells were heated in the absence of CADD522 in temperature-gradient thermocycler (38~60°C) for 10 min. After cooling, the samples were centrifuged to separate soluble fractions from precipitated proteins. Western blotting was performed for the β subunit using a specific antibody. DMSO, vehicle control (0.1%); β-F1, β-F1-ATP synthase. Right: thermal melting profile of the β-F1-ATP synthase. Band density of the the β subunit was quantified, normalized to loading (37°C), and analyzed for CETSA melting curves. Experiments were performed in three replicates (mean ± SD) and repeated twice. (**B**) Left: representative immunoblot result of the β-F1-ATP synthase upon increasing concentrations of CADD522. Right: dose-response target engagement of CADD522 in the β-F1-ATP synthase was performed at 58.2°C. Density of the the β subunit was quantified, normalized to the vehicle control (0.1% DMSO), and analyzed for melting curves.

Cellular thermal shift assay (CETSA) is used to assess drug: protein interactions based on the drug-induced thermal stabilization of target proteins (23828940, 29957028). Therefore, CETSA profiles a characteristic fingerprint of target engagement along the drug concentration axis [62, 63]. To delineate possible engagement of CADD522 with the F1-ATP synthase, we performed CETSA using specific antibodies to detect α- or β-F1-ATP synthase subunits (α-F1 & β-F1). The thermal melting profile of β-F1-ATP synthase in cell lysates with vehicle control (0.1% DMSO) revealed that at 58.2°C (= T_agg_[75]) and 53.5°C (= T_agg_[50]), 75% and 50%, respectively, of the β-F1-ATP synthase were aggregated and removed from solution (Figure 6A). To investigate drug concentration effects, we exposed cell lysates to high (0.14~100 μM) (Figure 6B) or low concentrations (0.00064~10 μM) of CADD522 (Supplementary Figure 8A) at a fixed heating temperature of T_agg_(75) (= 58.2°C). Band density of the β subunit increased as CADD522 concentration increased, suggesting that CADD522 stabilizes the protein upon heat challenge. Isothermal dose-response curves showed that 50% of the β-F1-ATP synthase remained stable with CADD522 at concentrations (EC50) of 1.60 μM (experiment 1) and 1. 94 μM (experiment 2). The T_agg_(75) and T_agg_(50) of the α-F1-ATP synthase in lysates with vehicle control were similar to those of the β-F1-ATP synthase (57.5°C and 52.0°C, respectively) (Supplementary Figure 8B). CADD522 increased stability of the α-F1-ATP synthase upon heat challenge (Supplementary Figure 8C). The EC50 of CADD522 for the α-F1-ATP synthase was lower than 1 μM (< 0.14 μM). These results indicate that CADD522 may interact with both α- and β-F1-ATP synthase. To confirm binding of CADD522 with F1-ATP synthase, we performed differential scanning fluorimetry (DSF), which monitors thermally-induced protein denaturation [64]. For this, we used purified, recombinant α- or β-F1-ATP synthase instead of crude lysates. Results showed that CADD522 (25~200 μM) increased significantly the melting temperature (Tm) of the purified α-F1-ATP synthase (Supplementary Figure 8D, left), indicating that CADD522 increased stability of the α subunit from heat denaturation. However, no significant change in the Tm of the recombinant β subunit was observed (Supplementary Figure 8D, right).

### CADD522 further enhances ROS levels under conditions of oxidative stress

ROS such as superoxide are converted to H_2_O_2_ within cells by superoxide dismutases [65, 66], and thus, a change in H_2_O_2_ can reflect a general change in the ROS level. Before evaluation of the effect of CADD522 on H_2_O_2_ levels, we first added CADD522 into Complete M without cells and incubated for 6 hr to verify that CADD522 itself does not generate ROS in culture medium. We determined the level of ROS using a commercial kit that generates luminescent signals (Luc) proportional to H_2_O_2_ concentrations. As shown in Supplementary Figure 9A (left), CADD522 did not significantly change H_2_O_2_ Luc compared to the vehicle control.

Cells treated with CADD522 for 6 hr also did not show a significant difference in the intensity of H_2_O_2_ Luc compared to the vehicle control (Supplementary Figure 9A, middle), which was inconsistent with intracellular ROS levels determined by CM-H_2_DCFDA (Figure 4A). This may be because of differences in assay sensitivity. However, CADD522 treatment for 18 hr significantly increased the intensity of H_2_O_2_ Luc in MDA-231 and MDA-468 cells, although the intensity was still low (< 100) (Supplementary Figure 9A, right).

**Figure 7:**
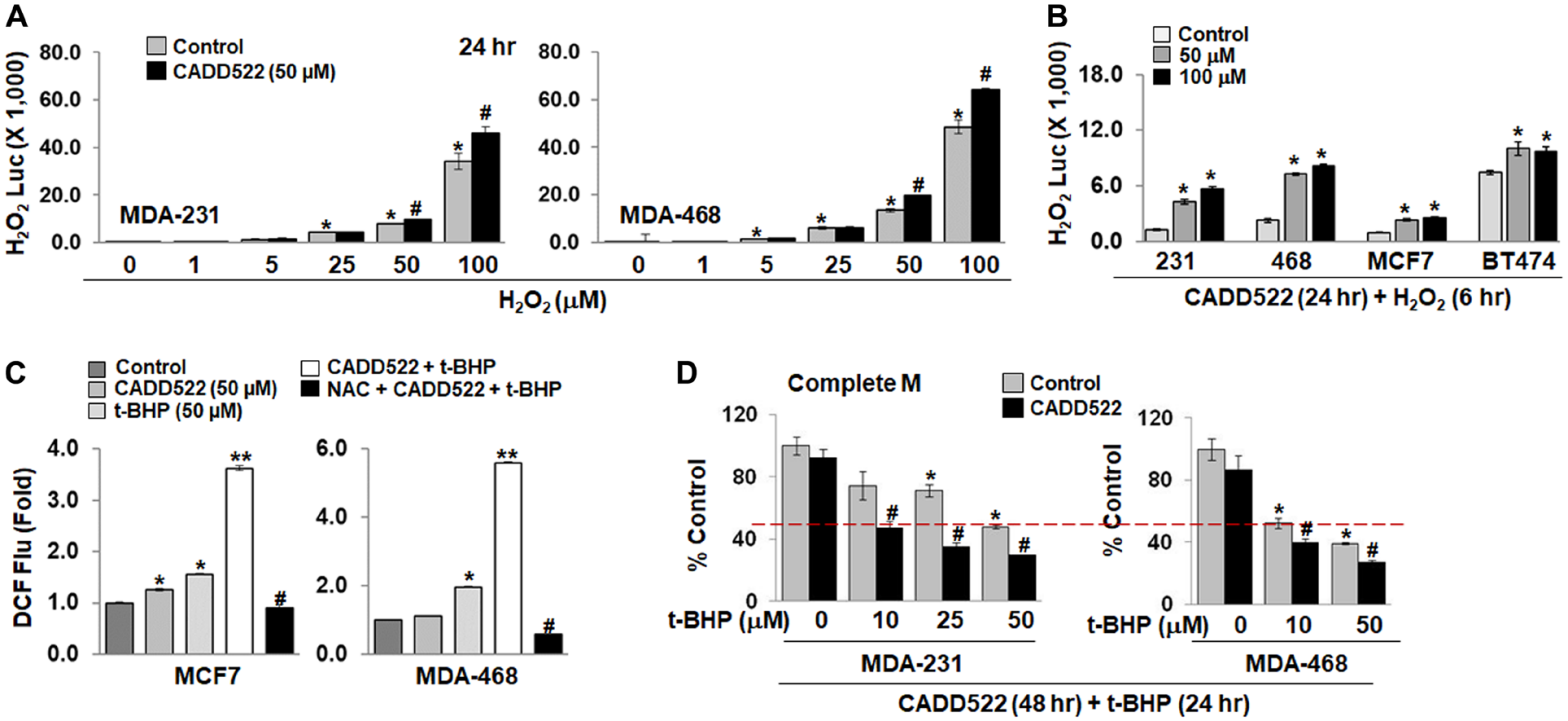
Enhanced ROS levels in the presence of ROS generating agents. (**A**) Cells in Complete M were treated with CADD522 (50 μM) in the presence of increasing concentration of H_2_O_2_ (0~100 μM) for 24 hr. The H_2_O_2_ Luc intensity was determined as described in Materials and Methods. Note, The H_2_O_2_ Luc intensity indicates Values at Y-axis × 1,000. Experiments were performed in triplicate and repeated twice (mean ± SD). ^*^ *P < 0.05* compared to Control with no H_2_O_2_ treatment; ^#^ *P < 0.05* compared to Control at indicated H_2_O_2_ concentrations. (**B**) B cells were treated with CADD522 for 18 hr, and then coincubated with H_2_O_2_ for additional 6 hr. ^*^ *P < 0.05* compared to Control. (**C**) Intracellular ROS levels were determined in MCF7 and MDA-468 cells after cells were treated with CADD522 and/or t-BHP for 4 hr in SFM. NAC (5 mM) was pretreated for 1 hr and further treated with CADD522 and t-BHP. Data are presented as the relative value to Control (Fold). Experiments were done in four replicates and repeated twice (mean ± SD). ^*^ *P < 0.05* compared to Control; ^**^ *P < 0.001* compared to t-BHP alone; ^#^ *P < 0.001* compared to CADD522 + t-BHP. (**D**) MDA-231 or MDA-468 cells were pretreated with CADD522 for 24 hr and further treated with t-BHP for 24 hr in the presence of CADD522. Cell growth was determined by crystal violet staining. ^*^ *P < 0.05* compared to no treatment; ^#^ *P < 0.05* compared to Control at indicated t-BHP concentrations.

Exogenous H_2_O_2_ (25 μM) generated a high level of H_2_O_2_ Luc intensity in cell culture medium without cells, and the intensity decreased in the presence of MDA-231 cells (Supplementary Figure 9B), which may be due to ROS quenching by cellular antioxidants or catalase. However, co-treatment with CADD522 and H_2_O_2_ for 6 hr further increased the H_2_O_2_ Luc intensity in MDA-231 cells. Similar results were observed in other BC cells co-treated with CADD522 and H_2_O_2_ for 6 hr (Supplementary Figure 9C) and 24 hr (Figure 7A), and BC cells pre-treated with CADD522 for 18 hr and then co-treated with H_2_O_2_ for an additional 6 hr (Figure 7B). Notably, the H_2_O_2_ Luc intensity increased as the concentration of exogenous H_2_O_2_ increased, which was further increased in the presence of CADD522 (Figure 7A).

*tert*-Butyl hydroperoxide (t-BHP) is an organic peroxide that induces oxidative damage and cell death [67]. To circumvent ROS scavenging by serum, we treated MCF7 and MDA-468 cells with CADD522 and/or t-BHP in serum-free medium (SFM) for 4 hr. Intracellular ROS levels were increased significantly by t-BHP alone (Figure 7C, Supplementary Figure 10A), and considerably enhanced by combined CADD522 and t-BHP treatment (Figure 7C). NAC (5 mM) robustly reduced the augmented ROS levels in both cell lines. A single treatment of CADD522 or t-BHP in SFM for 4 hr did not induce acute cell death in MCF7 and MDA-468 cells (cell viability ≥ 70%), whereas the combined treatment significantly decreased the viability (≤ 40%) (Supplementary Figure 10B). NAC completely blocked the combined treatment-induced cell death. Similar results were observed in MCF7 or MDA-468 cells co-treated in SFM for 24 hr, and in MDA-231 cells in Gal M (4 hr or 24 hr) (Supplementary Figure 10C).

Furthermore, when MDA-231 cells were grown in Complete M for 24 hr, CADD522 had little effect on the t-BHP-mediated cell growth inhibition (Supplementary Figure 10D, middle). However, pre-treatment with CADD522 for 24 hr before co-treatment significantly increased the t-BHP-mediated cell growth inhibition (Figure 7D), which was similar to that in cells treated simultaneously for 72 hr (Supplementary Figure 10D, right). Specifically, when MDA-231 cells were treated concomitantly for 72 hr, the cell growth at 10 μM t-BHP was 72.34 ± 4.25% in the vehicle control and 47.26 ± 6.28% in CADD522-treated cells. When cells were pre-treated with CADD522 for 24 hr and consecutively co-treated with t-BHP for additional 24 hr, the cell growth at 10 μM t-BHP was 74.43 ± 9.02% in vehicle control and 47.18 ± 4.41% in CADD522 treatment.

Menadione (Vitamin K3, a precursor of Vitamin K) is known to act as an anticancer agent in BC via the mitochondria-mediated apoptotic pathway [68, 69]. Menadione induces ROS and rapidly lowers ATP in MCF7 cells [68] and MDA-231 cells [70] through intracellular redox cycling [70]. The IC50 (50% inhibitory concentration) of Menadione in MCF7 and MDA-231 cells was reported to be 15 μM and 9 μM, respectively [68, 70]. To evaluate if Menadione potentiates the antiproliferative effects of CADD522 on BC cells, we treated MCF7 and MDA-231 cells with Menadione at a sublethal concentration (5 μM) in the presence or absence of CADD522 (0~100 μM) for 72 hr. CADD522 alone exerted higher growth inhibition in MCF7 cells than MDA-231 cells as reported [52], but MDA- 231 cells showed greater sensitivity to Menadione (57.82 ± 3.34%) than MCF7 cells (78.22 ± 3.50%) (Supplementary Figure 10E). In the presence of CADD522, the sensitivity of MDA-231 and MCF7 cells to Menadione increased significantly, which was more discernible in MDA-231 cells than in MCF-7 cells. The growth of MDA-231 cells was 31.61 ± 0.90% and that of MCF7 cells was 47.45 ± 3.79% in the presence of 50 μM CADD522. These data support the conclusion that CADD522, through its ability to elevate cellular ROS, is more effective at inhibiting proliferation when additional ROS-generating systems are provided.

### NAC attenuates the inhibitory effect of CADD522 on BC cells growth

**Figure 8:**
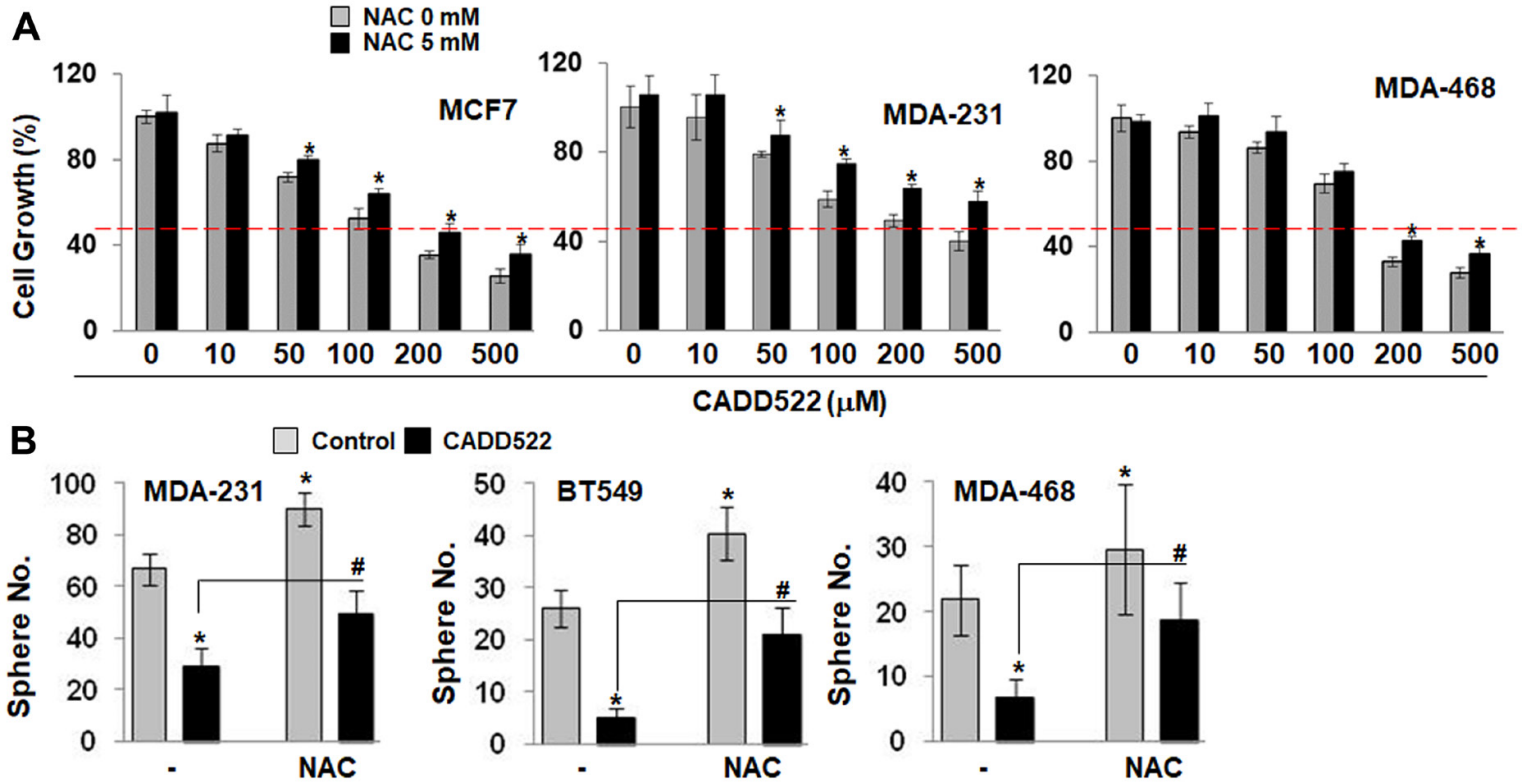
NAC protects BC cells from CADD522-mediated growth inhibition. (**A**) Cells were pre-treated with NAC (5 mM, pH adjusted to 7.2) for 1 hr, and cotreated with CADD522 (50 μM) for 72 hr. Cell growth was determined by crystal violet staining. Data are presented as mean ± SD. Experiments were done in triplicate and repeated twice. ^*^ *P < 0.05* compared to cells without NAC at indicated CADD522 concentrations. Red line, 50% of cell growth. (**B**) Cells were plated in low attachment plates and cotreated with NAC and CADD522 at the initial day of cell plating. Cells were incubated in suspension for 7 days. Data presented as mean ± SD. Experiments were done in triplicate and repeated three times. ^*^ *P < 0.05* compared to Control; ^#^ *P < 0.05* compared to cells treated with CADD522 in the absence of NAC.

Pre-treatment of tumor cells with NAC, a general ROS scavenger has been shown to provide protection against the cytotoxicity of intracellular oxidants [58, 71]. It is known that NAC can inhibit apoptosis [57] induced by ROS. To address the role of ROS in CADD522-mediated cell growth inhibition, we pre-treated BC cells with the antioxidant NAC (5 mM, pH adjusted to neutrality) for 1 hr and then co-treated with CADD522 (0~500 μM) for 72 hr. NAC moderately but significantly attenuated the CADD522-mediated cell growth inhibition (Figure 8A), indicating that the inhibition is partially ROS-dependent. In addition, NAC attenuated the CADD522-inhibited tumorsphere formation of BC cells (Figure 8B). The number of tumorspheres in cells treated with NAC in the presence of CADD522 (NAC+CADD522) was similar to that of the vehicle control, and importantly, the size and shape of the tumorspheres in cells with NAC+CADD522 were not disrupted compared to the vehicle controls without NAC (Supplementary Figure 11A). These results indicate that CADD522 may exert its antitumor activity at least through increased ROS levels and oxidative cell damage.

### Effect of CADD522 and RUNX2 KD on expression of mitochondria-associated genes

To begin to explore the molecular mechanisms that underlie how CADD522 or RUNX2 KD regulates mitochondrial function, we first determined mRNA levels of mitochondrial ETC and ATP synthase-encoding genes by quantitative PCR analysis. Results show that, overall, mRNA levels of these genes were not considerably different between RUNX2 KD and NTC of Hs578t cells or between CADD522-treated BC cells and the vehicle control (Supplementary Figure 12A–12D). One exception was the mRNA level of ATP5B (mitochondrial ATP synthase F1 subunit beta, β-F1-ATP synthase), which was markedly decreased by CADD522 in MDA-468 and MCF7 cells. RUNX2 KD significantly increased the level of PGC-1α, the master regulator of mitochondrial biogenesis, but had no effects on the levels of nuclear genes that regulate mitochondrial DNA transcription and replication (Supplementary Figure 12E). On the contrary, CADD522 significantly inhibited mRNA levels of the majority of mitochondrial biogenesis-related genes examined (Supplementary Figure 12F). Notably, the expression level of PGC-1α mRNA in MCF7 cells was almost completely suppressed by CADD522.

## DISCUSSION

Some BC cells expressing RUNX2 exhibit altered metabolic requirements, with reduced mitochondrial respiration and increased glycolysis [52, 54]. Under conditions where cellular ATP production is dependent on mitochondrial OXPHOS (Pyruvate M or Galactose M), ATP levels were lower in T47D-RUNX2 and MCF7-RUNX2 cells compared to their Empty controls (Figure 3B and 3C). However, in Serum-Free Glucose M in which mitochondrial ATP production was suppressed, ATP levels increased slightly but significantly in ectopic RUNX2-expressing cells (Supplementary Figure 4B, left), which may be due to RUNX2-mediated increases in glycolysis that result in increased ATP synthesis [52]. In complete M, ATP levels did not change (Supplementary Figure 4B, right). The ATP synthase activity also decreased in ectopic RUNX2-expressing cells compared to their Empty controls (Figure 5B and 5C, Supplementary Figure 6A and 6B, Supplementary Table 2).

Our results identify CADD522 as a novel OXPHOS inhibitor. The maximal respiratory capacity (MRC) and the reserve capacity (RC) between ectopic RUNX2-expressing cells and their Empty controls were not different (Supplementary Figure 2E), but CADD522-mediated inhibition of MRC and RC was higher in MCF7-RUNX2 cells than -Empty cells. These results suggest that RUNX2 might render BC cells more sensitive to CADD522. In addition, inhibition of OCR with CADD522 treatment was observed in MCF7 and T47D cells expressing either ectopic RUNX2 protein or low levels of baseline RUNX2. Decreased ATP levels were also observed in several BC cell lines regardless of their RUNX2 status. Moreover, intracellular and mitochondrial ROS levels increased in CADD522-treated ectopic RUNX2-expressing T47D and MCF7 cells (Supplementary Figure 5A and 5B) as well as Hs578t cells with RUNX2 KD. These findings suggest that the inhibitory effects of CADD522 on mitochondria are RUNX2-independent.

Recent findings indicate that glycolysis and mitochondrial OXPHOS cooperate under hypoxic and nutrient-deprived selective pressures for cancer cell growth. Under poorly perfused conditions, stem-like cancer cells that rely on mitochondrial OXPHOS for their survival exhibit drug resistance and increased metastatic potential [72]. Tumors that exist in nutrient-poor environments are not highly proliferative but continue to survive [73, 74] and, consequently, drugs that block mitochondrial ATP production are predicted to induce cell death in poorly perfused tumors. Tumors that show a heavy dependence on OXPHOS for ATP production (*i.e.,* cancer stem cells) appear to be more sensitive to drugs that limit mitochondrial ATP production since these tumors do not compensate for reduced mitochondrial ATP by increasing glycolytic ATP [42, 75–77]. Our published data suggests that blockage of RUNX2 with CADD522 inhibits glycolytic gene expression and reduces glucose uptake [52]. Our current results show that CADD522 decreased ATP production in cells under conditions of limited glucose availability. Therefore, a dual mechanistic basis for CADD522 action in BC may be operative: RUNX2-mediated glycolysis inhibition through suppression of Glut-1 gene expression [52] and RUNX2-independent mitochondrial dysfunction through repression of the activity of mitochondrial ATP synthase, an alternative target for CADD522. Our data showing that CADD522 and RUNX2 KD additively enhanced inhibition of cell growth and colony forming ability of BC cells (Supplementary Figure 11B and 11C) are supportive evidence for this concept. Moreover, CADD522 can synergize with therapies that diminish glycolysis [20, 78]. Indeed, we observed that concurrent treatment of CADD522 with 2-deoxyglucose (2-DG, an inhibitor of glucose uptake) significantly increased the sensitivity of MDA-231 cells to CADD522 (Supplementary Table 3). Therefore, CADD522 demonstrates mechanistic proof of concept by targeting mitochondrial ATP production alone and in combination with glycolysis inhibitors.

Oligomycin inhibits ATP synthase activity because it blocks proton (H+) translocation across the mitochondrial inner membrane (Fo subunits). Rotation of the central stalk against the surrounding α(3)β(3) subunits leads to synthesis of ATP at three separate catalytic domains on the β subunits [79, 80]. Our results are evidence that CADD522 inhibits the ATP synthase activity in both cell culture and cell-free lysates. The enzymatic assay used in our study measures the F1-mediated ATP hydrolysis activity (ATPase) as a surrogate for ATP synthase activity. In this *in vitro* system, the FoF1-ATP synthase/ATPase cannot use the mitochondrial protonmotive force (Δp) for ATP synthesis as there is no membrane gradient after immunocapture. The FoF1-ATP synthase thus does not exhibit Fo-dependent, proton-coupled enzymatic activity. Therefore, the F1 components of the ATP synthase are likely potential CADD522 targets.

Indeed, we identified the α and β subunits of the F1-ATP synthase as potential CADD522 interacting proteins, supporting that both α and β subunits of the F1-ATP synthase may be the actual CADD522 targets. In both CETSA and DSF, which use total protein lysates and purified individual proteins, respectively, CADD522 increased the protein stability of the α-F1-ATP synthase against heat challenge. CADD522 increased the stability of the β-F1-ATP synthases in total lysates, whereas it could not protect the purified, recombinant β-F1-ATP synthases from heat denaturation. These results suggest that CADD522 may directly interact with the α subunit, but indirectly with β subunit. Many F1-ATP synthase complex inhibitors interact with α and/or β subunits, but most common interaction sites for these inhibitors are known to be at the three α/β interface sites of the F1 complex [43]. With recombinant F1-ATP synthase components, we will validate in future studies whether individual α and β subunits or the α/β interaction sites are the relevant target, and how changes in α and/or β stability could account for CADD522 effects on mitochondrial ATP synthase activity [43]. Highly specific, cutting edge drug-protein interaction assays such as *in vivo* surface plasmon resonance (SPR) [81, 82] will be helpful to uncover at which sites of the F1-ATP synthase complex the functional binding occurs.

We reported that CADD522 promotes growth arrest rather than apoptosis [52]. Apoptosis can be activated with the use of compounds targeting key protein components of the mitochondria such as Bcl-2 family proteins (pro-survival Bcl-2 and Bcl-xL and pro-apoptotic Bax and Bak) [83]. The activity of OXPHOS is required for the function of the proapoptotic protein Bax in yeast and mammalian cells [42, 76, 84], which is dependent upon the mitochondrial FoF1-ATP synthase proton pump [77]. Oligomycin A, an inhibitor of Fo-portion of the H^+^-ATP synthase, halts the efficient execution of apoptosis [84, 85] and suppresses TNF-induced apoptosis [86]. This suggests that the activity of mitochondrial OXPHOS may be also required for the overall apoptotic potential of BC cells [86–89]. Therefore, the inhibitory effect of CADD522 on the activity of the FoF1-ATP synthase could be one of the reasons that CADD522 does not induce apoptotic changes in BC cells [52].

Metformin lowers ATP production through inhibition of mitochondrial ETC complex I [90]. Unlike metformin, CADD522 diminished ATP levels through inhibition of mitochondrial ATP synthase activity, without alteration of complex I activity. Oligomycin inhibits mitochondrial ATP synthase, which leads to changes in MMP (ΔΨm) [79, 80]. However, CADD522 had no effect on changes in the ratio of red to green fluorescence intensity of JC-1, indicating little changes in the overall MMP (ΔΨm). Targeting glycolysis and OXPHOS may lead to tissue toxicity. However, our previous report demonstrated the translational potential of CADD522 in *in vitro* and *in vivo* tumor models without exhibiting *in vitro* and *in vivo* toxicity [52]. Consistently, we did not observe significant difference in cell viability between CADD522- and vehicle-treated groups even when OCR and mitochondrial ATP synthase activity were substantially inhibited. Therefore, CADD522 may be uniquely different from other ATP synthase inhibitors (such as oligomycin) that demonstrate cytotoxicity and thus have no clinical application.

ROS production increases intracellular signaling and tumorigenesis but also is deleterious to cells, depending on the level and temporal expression of ROS, which may be cell-type specific [91–93]. Similarly, antioxidants have been shown to exhibit either pro-tumorigenic or anti-tumorigenic activity [92, 94, 95]. Many drugs target mitochondrial ATP synthase activity to lower ATP levels and increase ROS within mitochondria [15, 22]. Benzodiazepine (Bz-423) [96] and oligomycin A [97, 98] generate ROS from the respiratory chain by inhibition of mitochondrial ATP synthase. Therefore, the suppressed ATP synthase activity could contribute to the CADD522-mediated increase of ROS formation in mitochondria. CADD522 itself does not generate ROS but elevates ROS in cell-based systems. A combination of CADD522 with pro-oxidants that simultaneously increase ROS production might be more useful to limit cancer cell growth. For example, the combination of CADD522 with t-BHP or menadione further enhanced ROS levels and growth inhibition of MDA-231 cells. Our finding that N-acetyl cysteine (NAC) attenuates the CADD522-inhibited tumorsphere formation and cell proliferation in BC cells indicate that ROS might be responsible for CADD522-induced cell growth inhibition. Therefore, an increase in ROS could provide a therapeutic advantage if CADD522 also inhibits glycolysis through RUNX2 inhibition [52].

Oxygen consumption rate (OCR) is a feature of oxidative phosphorylation while the extracellular acidification rate (ECAR) usually depends on glycolysis. However, we did not observe a significant CADD522-induced change in ECAR (mpH/min/μg protein) in any cell lines tested. This may be due to buffering by HEPES (4-(2-hydroxyethyl)-1-piperazineethanesulfonic acid) that we freshly added to Seahorse-certified medium just before the assays. Addition of HEPES is known to provide a buffering capacity to cell culture medium to maintain physiological pH (7.2~7.6) despite changes in CO_2_ concentration produced by respiration. When cells are outside of a CO_2_ incubator to be processed on the XF Extracellular Flux Analyzer, HEPES can work as a “Good” buffer [99], thus maintaining neutral pH even under conditions that increase ECAR with inhibition of mitochondrial respiration.

The mitochondrial metabolic properties of cancer cells are different from those of normal cells. In contrast to normal cells, actively growing tumor cells exhibit increased mitochondrial biogenesis and respiration for ATP generation to meet energy demands and maintain survival [100, 101]. Cancer stem cells are slow-growing cells that harbor tumorigenic potential, self-renewal capabilities, and intrinsic resistance to conventional and targeted therapies. These cells are dependent on active mitochondria and show increased oxygen consumption and ATP production compared to more highly proliferating tumor cells [100, 102–104]. Therefore, inhibition of the mitochondrial respiratory chain can be exploited to develop selective anticancer agents [26], since drugs targeting the ETC can preferentially kill cancer cells [22]. We reported that cell growth inhibition by CADD522 was significant in 13 different BC cells (TNBC, ER^+^, and HER2^+^), while resistance of normal epithelial cells to CADD522 was commonly observed [52]. Moreover, tumorspheres organized from stem-like BC cells in suspension culture (MCF7, MDA-231, MDA-468) were sensitive to CADD522 treatment, but MCF10A cells that resemble normal mammary epithelial cells (mammospheres) were resistant [52]. Therefore, the higher sensitivity of BC cells to CADD522 compared with normal epithelial cells might be due to inhibitory effects of CADD522 on mitochondrial OXPHOS and the increased ROS levels.

Mitochondrial biogenesis is the process via which cells increase their individual mitochondrial mass [105]. RUNX2 KD in Hs578t cells increased the mitochondrial mass, which was reflected by increased fluorescence intensity of MitoTracker Green dye, and the mRNA level of PGC-1α, indicating that RUNX2 might be involved in regulation of mitochondrial biogenesis. CADD522 treatment for 12 hr did not change mRNA levels of mitochondria-encoded genes (CytoB, MT-ATP6, MT-CO2, etc.). However, CADD522 significantly decreased the mRNA levels of nucleus-encoded mitochondrial genes (PGC-1α, NRF-1, TFB2M, *etc.*) that activate the expression of key metabolic genes regulating respiration and mitochondrial DNA transcription and replication. Therefore, we do not exclude the possibility that CADD522 might interfere with mitochondrial dynamics through regulation of nuclear genes whose products target the processes that regulate mitochondrial content, structure, and function. Further studies will be needed to verify the precise effects of CADD522 on regulation of mitochondrial biogenesis.

Moreover, expression of the catalytic subunit β-F1-ATP synthase is tightly regulated by post-transcriptional mechanisms that affect mRNA localization, stability and translation [106–110]. The mRNA expression of ATP5B in MCF7 and MDA-468 cells was substantially decreased by CADD522 treatment. These results indicate that CADD522 might inhibit ATP synthase activity through regulation of target gene expression levels and/or the activity of the catalytic β subunit of the mitochondrial F1-ATP synthase. In summary, our findings demonstrate that mitochondrial ATP synthase inhibition may be a valid therapeutic approach and that one mechanism that drives sensitivity of BC cells to CADD522 is suppression of mitochondrial metabolism.

## MATERIALS AND METHODS

### Cell lines and reagents

All BC cell lines were obtained from American Type Culture Collection. Cells were subjected to routine cell line quality examinations (*e.g.,* morphology, Mycoplasma) every 6 months. The cells for experiments were passaged for less than 6 months. Cell lines were maintained in DMEM/F12 (50:50) (BT474 and MDA-MB-468), RPMI1640 (HCC1937, HCC1428, BT549, HCC70), McCoy-5A (SKBr3), and DMEM (all other BC cancer cells) supplemented with 10% FBS, 100 U/mL penicillin and 100 mg/mL streptomycin. Establishment of T47D and MCF7 cells stably expressing ectopic RUNX2 (T47D-RUNX2 and MCF7-RUNX2), and their empty vector controls (T47D-Empty and MCF7-Empty) were previously described [52]. These cells were maintained under G418 (0.5~1 mg/ml). Hs578t cells with RUNX2 knockdown (KD) (55.5), a negative clone of RUNX2 KD cells (54.5) and non-targeting control cells (NTC) were previously described [52, 54]. These cells were maintained under puromycin (1 μg/ml). Notably for experiments, to avoid unwanted effects of G418 or puromycin on mitochondria, these cells were plated in cell culture medium without antibiotics and incubated for a day, and then processed immediately or treated with CADD522 in antibiotics-free medium. CADD522 was purchased from ChemBridge Corporation (San Diego, CA). Other drugs or agents were purchased from Sigma-Aldrich (St Louis, MO, USA). Small-Interfering RNA (siRNA) pool targeting RUNX2 and non-targeting control were purchased from Dharmacon (Lafayette, CO, USA), and transfected into cells using RNAiMAX Reagent (Invitrogen).

### Mitochondrial oxygen consumption rate (OCR)

Dynamic changes in OCR, a result of oxidative phosphorylation, were measured in live cells using the Seahorse XF24 Extracellular Flux Analyzer (Agilent Technologies, Santa Clara, CA, USA) according to the manufacturer’s instructions. BC cells were seeded (> 60,000 cells/well) in 24-well Seahorse plates and incubated in complete growth medium. The following day, cells were treated with CADD522, and incubated up to 72 hr. At the time of OCR measurement, cells were replenished with fresh Seahorse-XF DMEM (Cat. No. 102365-100) supplemented with 5 mM HEPES, 5 mM glucose and 1% serum (pH-adjusted to 7.4 with Tris base powder) in the absence of CADD522 and incubated for one hour at 37°C in a non-CO_2_ incubator for stabilization of temperature and pH. Cell plates were then loaded onto the Seahorse Analyzer equipped for the sequential addition of final concentrations of oligomycin (1 μg/ml), carbonylcyanide-4-trifluoromethoxyphenylhydrazone (FCCP, 1 μM), Pyruvate (10 mM) and Antimycin A (1 μM), with compounds that were freshly prepared just before use. In some experiments, we added FCCP and/or Pyruvate to two or three ports in the flux analyzer without oligomycin to maximize and prolong the respiratory reserve capacity measurement. At the end of the assay, total protein content in individual wells was determined and used for normalization of raw OCR values. OCR was expressed as pMoles/min/μg protein.

To determine the acute OCR response in separate experiments, T47D-RUNX2 and -Empty cells without prior CADD522 treatment were replenished with Seahorse medium an hour before the assay. At the time of the assay, the assay buffer with 0.1% DMSO, oligomycin or increasing concentrations of CADD522 (10~200 μM, final concentration) was injected into port A, and the OCR was measured at 37°C. Data are presented as OCR (%), the percentage of the basal OCR at t = 12 min.

Mitochondrial respiration (OCR) was calculated as maximal respiratory capacity (MRC) by subtracting non-mitochondrial respiration (NM, OCR value at t = 63 min) from the FCCP rate; ATP production-linked respiration (AP) by subtracting the oligomycin rate from baseline cellular OCR; proton leak-linked respiration (PL) by subtracting NM from the oligomycin rate; and mitochondrial reserve capacity (RC) by subtracting basal respiration (OCR value at t = 12 min) from MRC (Supplementary Figure 2, right). Baseline cellular OCR (BL) was calculated from basal respiration after subtracting NM [111]. Percent (%) inhibition of the individual parameters was calculated from the equation (A−B)/A × 100; A, vehicle control; B, CADD522 treatment.

### ATP assay

Cells were treated with CADD522 for the indicated time periods in pyruvate medium (Pyruvate M, 2 mM pyruvate, no glucose, no glutamine, 5% serum), galactose medium (Galactose M, 5 mM galactose, 1 mM pyruvate, no glucose, 2 mM glutamine, 10% serum), normal cell growth medium (Complete M, 25 mM glucose, 2 mM glutamine, 1 mM pyruvate, 10% serum), or Serum-Free Glucose M (25 mM glucose, no glutamine, no pyruvate, 0% serum), and ATP level was determined using Luminescent ATP Detection Assay Kit purchased from Abcam (Boston, MA) as per manufacturer’s instructions. After addition of the assay mixture containing luciferin and luciferase, luminescence was measured using a Wallace microplate luminescence reader (Perkin Elmer, Waltham, MA, USA).

### Intracellular reactive oxygen species (ROS) and mitochondrial superoxide levels

Cells in 96 well plates were treated with CADD522 in normal growth or serum-free medium (SFM). To detect the intracellular formation of ROS, cells were incubated with a fluorogenic dye 2′,7′-dichlorofluorescein diacetate (CM-H_2_DCFDA, 25 μM) (ThermoFisher Scientific) for 1 hr. This dye produces the highly fluorescent product 2′,7′-dichlorofluorescein (DCF) upon reaction with ROS. The fluorescence was measured at 488/535 nm (Ex/Em) using a SYNERGY/HTX multimode reader (BioTek). CM-H2DCFDA is more sensitive to oxidation by hydrogen peroxide (H_2_O_2_) than superoxide [112].

To detect mitochondrial superoxide levels, cells were incubated with MitoSox Red (5 μM) (ThermoFisher Scientific) in HBSS for 10 min and the fluorescence was measured at 510/580 nm (Ex/Em). In parallel, the cell viability of cells receiving the same treatments was determined using the cell-permeant dye Calcein-AM (2 μg/ml) (ThermoFisher Scientific). The nonfluorescent Calcein-AM is converted to green-fluorescent Calcein by intracellular esterases in live cells, which is measured at 495/515 nm (Ex/Em). DCF or MitoSox Red fluorescence was divided by Calcein green fluorescence to normalize the ROS signal for cell viability.

### Mitochondrial ATP synthase activity assay

Quantitative measurement of the activity of mitochondrial ATP Synthase was performed using the ATP Synthase Enzyme Activity Microplate Assay Kit that is commercially available (Abcam, ab109714). After cells were treated with CADD522 for the indicated period of time in Complete M, cells were harvested and cell lysates were extracted in buffers provided by the kit. ATP synthase is localized in the mitochondria and the plasma membrane of highly proliferating cells in eukaryotes [32, 50]. Therefore, homogenized samples (cell lysates) were frozen, thawed and pelleted by centrifugation at ~16,000 rpm to fracture the plasma membranes and remove soluble non-membrane associated proteins as per manufacturer’s instruction. Pelleted samples were resuspended in the supplied buffer. Enzymes in cell lysates (50 μl/well) were immunocaptured within the wells of the microplate and enzyme activity was determined by following the reduction of substrates, coupled to the reduction of a reporter dye to yield a colored reaction. Absorbance of each well was measured every 2 min for up to 90 min in a spectrophotometer purchased from BioTek Instruments (SYNERGY/HTX multimode reader) (Winooski, VT, USA) at a wavelength of 340 nm (kinetic program), and the slope (= − Enzyme Activity) was calculated from the linear range in each curve by the equation ([ΔAbsorbance or ΔRatio]/Δmin/μg protein in 50 μl × 1000).

For the *in vitro* mitochondrial ATP Synthase activity assay, 50 μg/50 μl of protein lysates isolated from MDA-231 and MDA-468 cells were directly incubated in the presence or absence of CADD522 (0~2 μM), and ATP Synthase Activity was measured. Relative activity is expressed as changes in absorbance or Ratio per minute ([ΔAbsorbance or ΔRatio]/Δmin/50 μg protein ×1000). Ratio indicates relative value to the absorbance at 0 min.

### Mitochondrial membrane potential (MMP) (ΔΨm)

The changes in MMP were evaluated using a cationic fluorescent indicator (JC-1; Molecular Probes, Eugene, OR, USA), which aggregates in polarized mitochondria (red fluorescence), indicating high or normal MMP and remains in monomeric form in the cytoplasm when mitochondria are depolarized (green fluorescence), indicated low MMP. Cells in 96 well plates were incubated in the growth medium containing 1 mg/mL JC-1 dye for 30 minutes at 37°C, and fluorescence was acquired at 488/525 nm (ex/em) for JC-1 monomers and 488/590 nm (ex/em) for J-aggregates using a fluorescence microplate reader (BioTek). MMP (ΔΨm) was expressed as the ratio of red to green fluorescence intensity. JC-1 aggregation is a survival marker and JC-1 monomer is a cell death indicator.

Alternatively, we assessed the MMP using tetramethylrhodamine methyl ester (TMRM), a cell-permeant, cationic, red fluorescent dye that accumulates in active (healthy and functioning) mitochondria with intact membrane potentials. Upon loss of the MMP, TMRM accumulation ceases and the signal disappears. Cells were incubated with TMRM (50 μM) for 10 min and fluorescence intensity was measured at 561/590 nm (ex/em) in a microplate reader (BioTek). Carbonylcyanide-3-chlorophenylhydrazone (CCCP, 50 μM), a mitochondrial uncoupler, was used as a positive control for the MMP reduction.

### Measurement of mitochondrial mass

Mitochondrial mass was determined with the MitoTracker Green FM (Life Technologies) dye that is non-fluorescent in aqueous solutions, but becomes fluorescent upon accumulating in the mitochondrial lipid environment regardless of membrane potential [113]. Cells plated in 96 well plates were treated with CADD522 in phenol red–free growth medium for 24 hr. Cells were washed with HBSS, and then incubated with Mitotracker Green as per manufacturer’s protocol. Green fluorescence was determined at 490/516 nm (ex/em) in a fluorescence microplate reader (BioTek).

### Complex I (NADH dehydrogenase) activity assays

Quantitative measurement of Complex I activity was performed with a commercially available Complex I Enzyme Activity Microplate Assay Kit (Abcam, ab109721) as per manufacturer’s instruction. Cell conditions for measuring Complex I activity were the same as described for measuring the mitochondrial ATP synthase activity. The activity was determined by measuring the oxidation of NADH to NAD+ with the simultaneous reduction of a dye (increased absorbance at 450 nm) for 30~60 min. Protein concentration was used to normalize activity (ΔAbsorbance /Δmin/μg protein in 50 μl × 1000), which was linear between 10–400 μg protein. Data are presented in arbitrary units (au).

### Cellular thermal shift assay (CETSA)

CETSA [62, 63] was performed with MDA-MB-231 cells cultured in DMEM medium supplemented with 10% FBS. For an initial determination of the melting profile of α- or β-F1-ATP synthase subunits (α-F1 and β-F1), fresh cell lysate prepared in non-denaturing buffer was dispensed into a 96-well PCR plate in the above medium (approx. 6,000 cells/50 μl/well), and then was subjected to temperature gradient (38~60°C) for 10 min. Subsequently, centrifugation was performed at 14.000 rpm to sediment the unstable protein content. Supernatant was collected and SDS-PAGE gel was run, and immunoblot analysis was performed for α-F1 and β-F1 using corresponding primary antibodies. Band intensity was quantified on C-Digit Blot Scanner (LI-COR Biosciences, Lincoln, NE, USA), and subsequently T_agg_(50) and T_agg_(75) values (temperatures at which 50% and 75% of the initial protein was reduced, respectively) were calculated for each protein. In a subsequent run, fresh lysates of MDA-MB-231 cells were treated at various doses of CADD522 and vehicle control (0.1% DMSO) for 1 hr. Samples were then subjected to heat shock at T_agg_(75) for 10 min, and unstable protein was removed by centrifugation step. Following an immunoblotting step, bands of stable target proteins were quantified, normalized to loading control and plotted using GraphPad Prism software. EC50 values of CADD522 with each target protein were calculated.

### Differential scanning fluorimetry (DSF)

Recombinant human mitochondrial ATP synthase subunit α (α-F1-ATP synthase) and β (β-F1-ATP synthase) were purchased from CusaBio (Houston, TX, USA). Binding of CADD522 to α or β subunit was examined experimentally using DSF, which evaluates changes in the target protein melting temperature (Tm) due to interactions with the test compound [64, 114, 115]. SYPRO orange (Thermo Fisher Scientific) diluted 1:1000 in 10 mM HEPES, 150 mM NaCl (pH 7.5), and 1.5 μM recombinant proteins were added to 96-well PCR plates. Then 0 to 200 μM CADD522 dissolved in DMSO (1% final DMSO concentration) was added, the plates were mixed, sealed, centrifuged at 1,000 rpm for 1 min, and melting curve analysis was performed using an Applied Biosystems StepOne real-time PCR instrument. The midpoint was determined from the first derivative curve, and the corresponding temperature (Tm) was taken from the midpoint.

### Hydrogen peroxide (H_2_O_2_)-ROS levels

Hydrogen peroxide (H_2_O_2_)-ROS levels were determined using a commercial kit (ROS-Glo™ H_2_O_2_ Assay kit, Promega), a luminescence-based assay that measures the level of H_2_O_2_ directly in cell culture. Luciferin and recombinant Luciferase in the kit generate a luminescent signal that is proportional to H_2_O_2_ concentration. Cells plated in 96 Black (or white) well plates and incubated in 10% DMEM (Complete M) were treated with CADD522 with or without H_2_O_2_ (25 μM) for 6~24 hr. After exposure to H_2_O_2_, 50 μl of media samples were transferred to a separate plate to combine with an equal volume of ROS-Glo™ Detection Solution, and the ROS-Glo™ H_2_O_2_ Assay was performed as per manufacturer’s instruction (the non-lytic assay). H_2_O_2_ substrate provided by the kit was exogenously added for ROS generation.

### Cell growth and survival assay

Cell growth was determined by crystal violet staining. Cells were plated on 96-well (30,000 cells/well) or 24-well plates (50,000 cells/well). After CADD522 treatment, cells were incubated for 24~72 hr. Cells were stained with crystal violet (0.5% in methanol: acetic acid = 3:1) and washed with PBS. Crystal violet was solubilized in DMSO and measured in a microplate reader at 592 nm. For tumorsphere growth, single cell suspensions (100,000 cells/well) were plated in 6-well ultra-low attachment plates (Corning) with 5 ml of DMEM supplemented with 10% serum. CADD522 (50 μM) was added at the day of the plating or 4 days after plating. Tumorspheres were photographed and counted at the final day of the assay (day 7). Spheres were counted from 9 fields per well and averaged from triplicate. For clonogenic survival determination, cells were plated on 6-well plates (200 ~500 cells/well). After CADD522 (50 μM) treatment, cells were incubated for 2~3 weeks without changing media. Colonies were fixed in Methanol-Acetic Acid solution (3:1) and stained with crystal violet (0.5%). After washing, colonies were photographed and counted.

### Quantitative real time-RT-PCR (Q-RT-PCR)

Total RNA was extracted using TRIzol (Life Technologies). One μg of total RNA was reverse transcribed with oligo-(dT) primer using the SuperScript first-strand synthesis system (Invitrogen) to synthesize cDNA. One μl of each cDNA was used for real-time RT-PCR using QuantiFast SYBR Green PCR Kit (Promega). mRNA expression of gene of interest relative to β-actin was calculated based on the threshold cycle (C_t_) as 2^-Δ(ΔCt)^ method. Primer sequences are listed in Supplementary Table 4.

### Statistical analysis

Results from cell culture assays are expressed as mean ± SD from at least three independent experiments. Comparisons of quantitative data between two groups were analyzed using the two-tailed Student’s *t*-test. For the *in vivo* study, data are expressed as mean ± SE. All multiple comparisons were followed by Tukey’s post-hoc adjustment following ANOVA and Mann-Whitney non-parametrical tests. All statistical analyses were conducted using STATA version 14 (STATA Inc., College Station, TX, USA). *P* values less than 0.05 were considered significant.

## SUPPLEMENTARY MATERIALS



## Abbreviations

BC: Breast cancer
OCR: Oxygen consumption rate
CADD: Computer-Assisted Drug Design
ROS: reactive oxygen species
OXPHOS: oxidative phosphorylation
PDH: pyruvate dehydrogenase
MMP: mitochondrial membrane potential
KD: knockdown
NTC: non-targeting control cells
MRC: maximal respiratory capacity
NM: non-mitochondrial respiration
AP: ATP production-linked respiration
BL: baseline cellular OCR
PL: proton leak-linked respiration
RC: mitochondrial reserve capacity
SFM: serum-free medium
CETSA: Cellular thermal shift assay
DSF: Differential scanning fluorimetry
ATP5A: mitochondrial ATP synthase subunit alpha (α-F1-ATP synthase)
ATP5B: mitochondrial ATP synthase subunit beta (β-F1-ATP synthase)
NAC: *N*-acetyl-l-cysteine
