# Supplementary material for: Targeting breast cancer metabolism with a novel inhibitor of mitochondrial ATP synthesis
Source: Oncotarget. 2020 Oct 27;11(43):3863–85. doi: 10.18632/oncotarget.27743 (PMC7597410; doi:10.18632/oncotarget.27743)
Supplement: Supplementary file 1 [file oncotarget-11-3863-s001.pdf]

# Targeting breast cancer metabolism with a novel inhibitor of mitochondrial ATP synthesis

## SUPPLEMENTARY MATERIALS

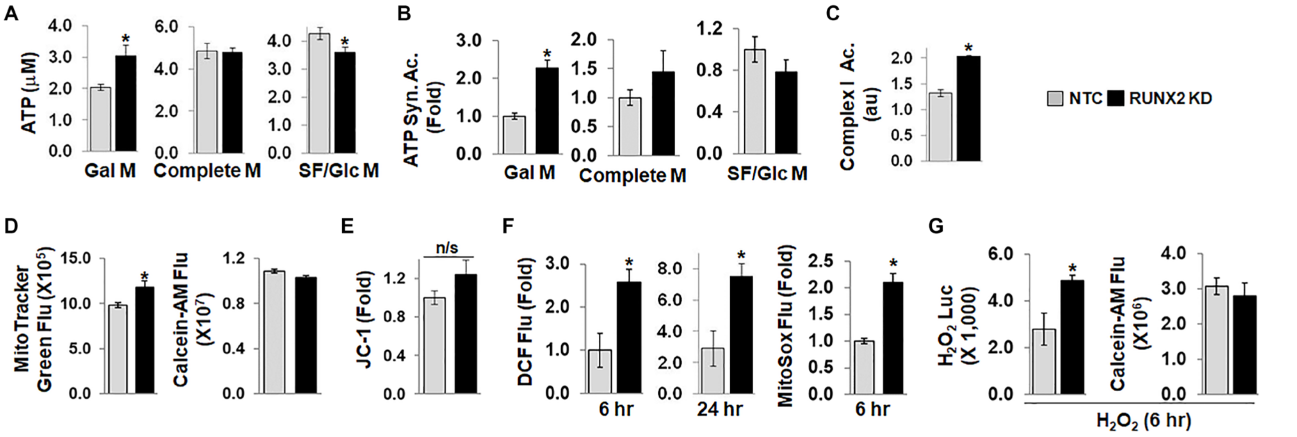


**Supplementary Figure 1: RUNX2 KD increases ATP synthesis.** Hs578t cells with RUNX2 KD and non-targeting control (NTC) were incubated in Galactose M (Gal M), Complete M, or Serum-Free Glucose M (SF/Glc M) for 4 hr, and ATP levels (**A**) and mitochondrial ATP synthase activity (ATP Syn. Ac.) (**B**) were determined as described in Materials and Methods. Experiments were performed in triplicate and repeated twice (mean ± SD). Fold-differences were calculated from the relative value to the average of the NTC (= 1). $^{*}P < 0.05$ compared to NTC. (**C**) Cells were incubated in Complete M for 24 hr, and Complex I activity were determined as described in Materials and Methods. Data are presented in arbitrary units (au). (**D**) Cells in Complete M were incubated for 24 hr, and mitochondrial mass was determined using the MitoTracker Green FM dye that accumulates in the mitochondrial lipid environment regardless of membrane potential [113]. Data are presented as fluorescence intensity of MitoTracker Green FM dye. Experiments were performed in triplicate and repeated twice (mean ± SD). $^{*}P < 0.05$ compared to NTC (left). Cell viability was assessed by Calcein-AM assay (right). Flu, fluorescence. (**E**) Hs578t cells with RUNX2 KD and NTC were incubated in Complete M for 24 hr, and MMP (ΔΨm) was determined by JC-1 dye as described in Materials and Methods. (**F**) Intracellular ROS (DCF fluorescence, left) and mitochondrial ROS levels (MitoSox fluorescence, right) were determined 6 and 24 hr after cells were incubated in Complete M. Data (DCF fluorescence relative to Calcein green fluorescence) are presented as Fold, which was calculated from the relative value to the average of the NTC (= 1). (**G**) Hs578t cells with RUNX2 KD and NTC were incubated with $H_2O_2$ for 6 hr, and $H_2O_2$ Luc intensity was measured. The $H_2O_2$ Luc intensity indicates Values at Y-axis X 1,000.

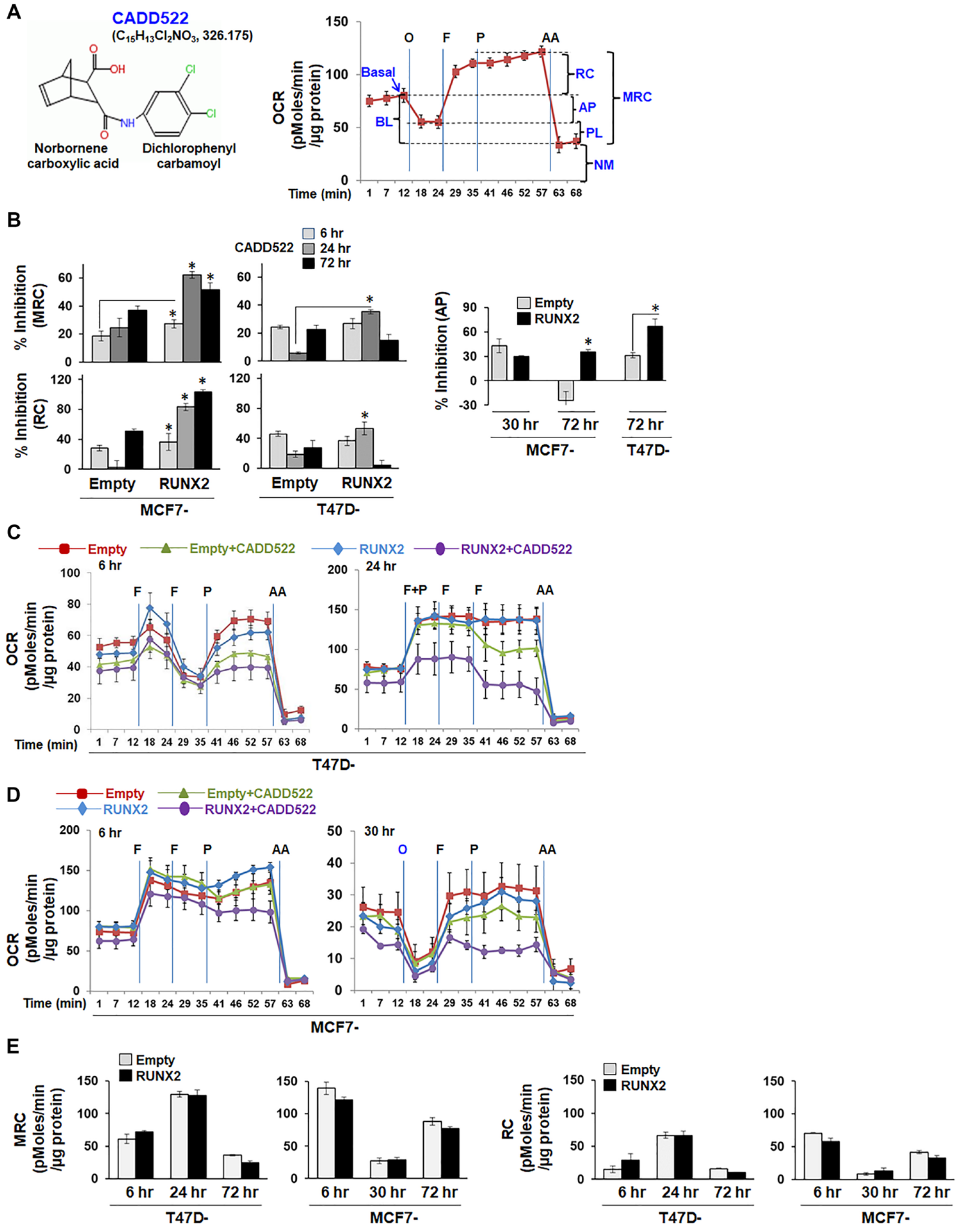


**Supplementary Figure 2:** (**A**) Chemical structure of CADD522 (left) and representative raw data trace (right). FCCP (F) was used to increase the inner mitochondrial membrane permeability to protons and allow maximum electron flux through the ETC, resulting in the maximum amount of oxygen consumption. Pyruvate (P, 10 mM) was added as an exogenous substrate to ensure sufficient substrate availability to support maximum respiration. ATP production-linked and proton leak-linked OCR were measured by the addition of oligomycin A at port A of the XF analyzer. As expected, the addition of oligomycin A (O) decreased the ATP-production-linked OCR but FCCP robustly increased the MRC and RC (Figure 1). Addition of antimycin A (AA), an irreversible complex III inhibitor, resulted in complete inhibition of mitochondria-dependent OCR in all tested groups. Mitochondrial respiration (OCR) was calculated as maximal respiratory capacity (MRC) by subtracting non-mitochondrial respiration (NM, OCR value at t = 63 min) from the FCCP rate; ATP production-linked respiration (AP) by subtracting the oligomycin rate from baseline cellular OCR; proton leak-linked respiration (PL) by subtracting NM from the oligomycin rate; mitochondrial reserve capacity (RC) by subtracting basal respiration (OCR value at t = 12 min) from MRC. Baseline cellular OCR (BL) was calculated from basal respiration after subtracting NM [111]. (**B**) Cells were treated with CADD522 (50 μM), and replenished with Seahorse-Certified Medium without CADD522. OCR was measured in the XF Extracellular Flux Analyzer. Data are presented as percent (%) inhibition of the individual parameters, which was calculated from the equation (A-B)/A X 100 (A, vehicle control; B, CADD522 treatment). Data are presented as mean ± SE from two independent experiments. $^{*}P < 0.05$ compared to % inhibition of the Empty cells at indicated period of time. (**C**, **D**) Cells were treated with CADD522 for 6 × 30 hr, and OCR was measured in the XF Extracellular Flux Analyzer. Experiments were performed in four replicates and repeated twice (mean ± SD). O, Oligomycin (1 μg/ml); F, FCCP (1 μM, port B); P, pyruvate (10 mM, port C); AA, Antimycin A (1 μM, port D). (**E**) MRC and RC in ectopic RUNX2-expressing T47D and MCF7 and their Empty cells 6~72 hr after cells were incubated in normal growth medium without G418. Experiments were performed in four replicates and repeated twice (mean ± SE).

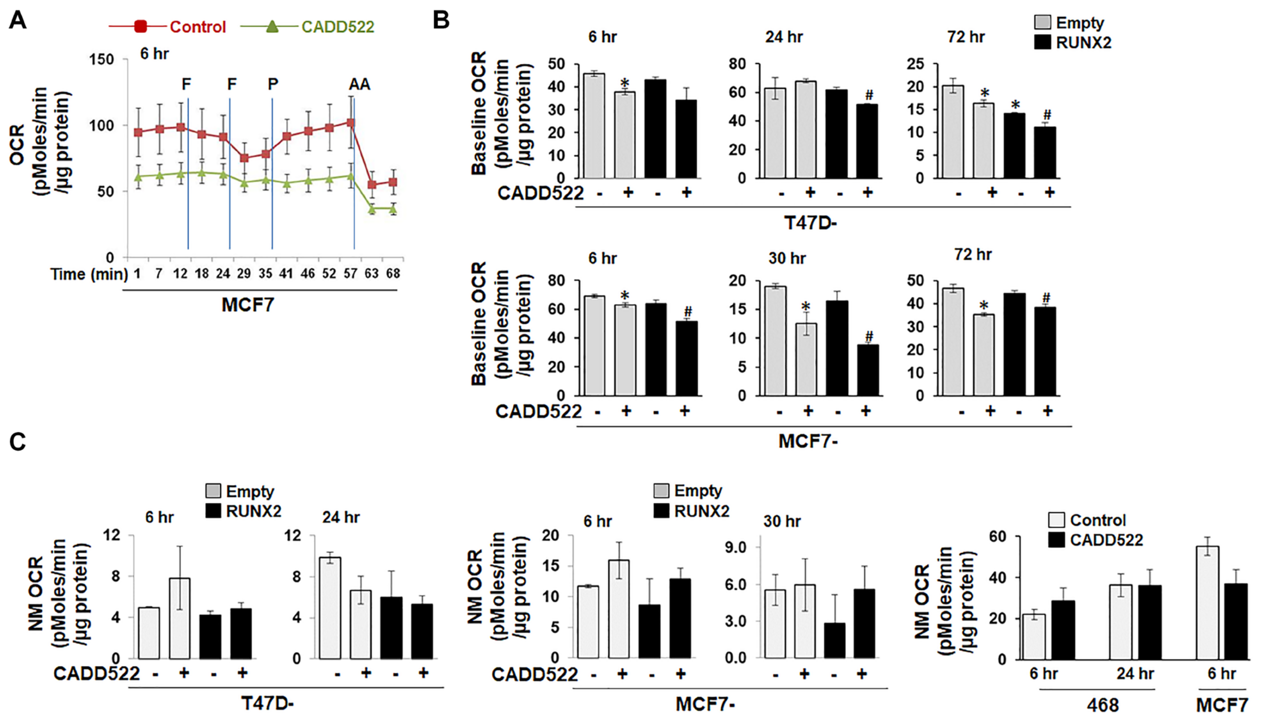


**Supplementary Figure 3:** (**A**) Cells were treated with CADD522 for 6 hrs, and OCR was measured in the XF Extracellular Flux Analyzer. Experiments were performed in four replicates and repeated twice (mean ± SD). O, Oligomycin (1 μg/ml); F, FCCP (1 μM, port B); P, pyruvate (10 mM, port C); AA, Antimycin A (1 μM, port D). (**B**) Baseline cellular OCR (BL) was calculated from basal respiration (value at t = 12 min) after subtracting NM. Experiments were done in four replicates and repeated twice (mean ± SE). $^{*}P < 0.05$ compared to the vehicle control (0.1% DMSO) of Empty Controls. $^{\#}P < 0.05$ compared to the control of RUNX2-expressing cells. (**C**) Non-mitochondrial respiration (NM, value at t = 63 min) between vehicle- and CADD522-treated cells. Results of CADD522 treatment for 72 hrs in RUNX2 expressing T47D and MCF7 cells and their Empty controls were shown in Figure 1C.

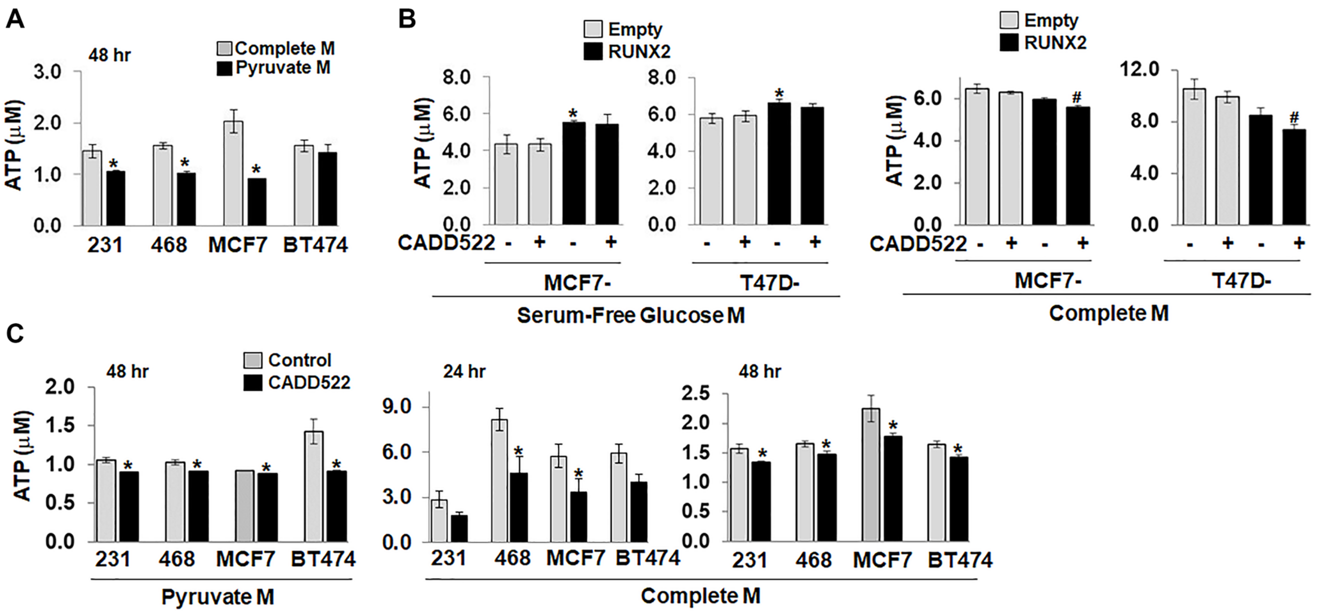


**Supplementary Figure 4:** Cellular ATP levels were measured in cells grown in Pyruvate M or Complete M for 48 hr without CADD522 treatment (**A**) or in cells with or without CADD522 (50 μM) for 24 or 48 hr (**C**). Experiments were performed in four replicates and repeated twice (mean ± SD). $^{*}P < 0.05$ compared to the vehicle control. (**B**) Ectopic RUNX2 expressing T47D and MCF7 cells and their Empty controls were treated with CADD522 for 6 hr in Serum-Free Glucose M (DMEM with 25 mM glucose, no glutamine, no pyruvate, 0% serum) (left) or Complete M (right), and cellular ATP levels were determined. $^{*}P < 0.05$ compared to the vehicle control of Empty cells; $^{\#}P < 0.05$ compared to the vehicle control of ectopic RUNX2-expressing cells.

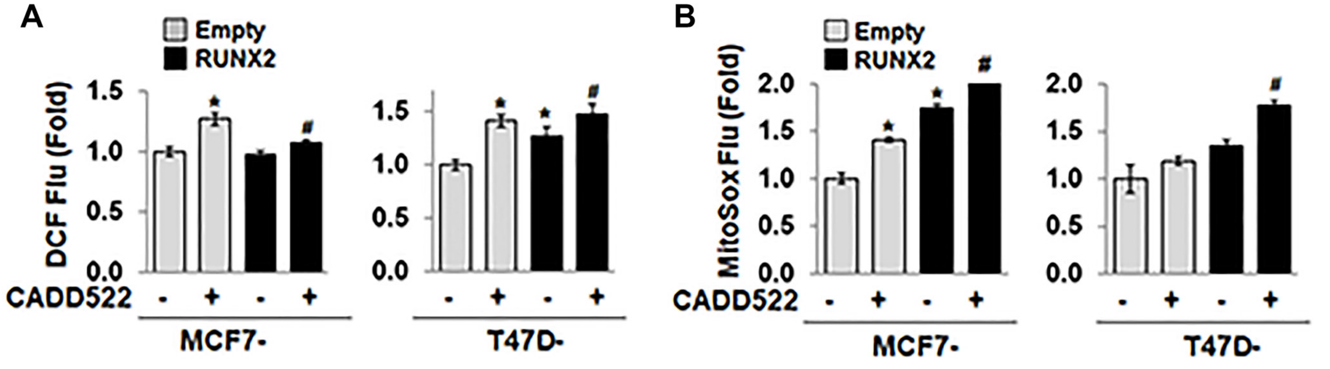


**Supplementary Figure 5:** Intracellular ROS (**A**) and mitochondrial superoxide levels (**B**) were determined in ectopic RUNX2 expressing T47D and MCF7 cells and their Empty controls. Cell were treated with CADD522 for 24 hr in normal growth medium. Similar results were observed in cells treated with CADD522 for 6 hr. Experiments were performed in four replicates and repeated twice (mean ± SD). $^{*}P < 0.05$ compared to the vehicle control of Empty cells; $^{\#}P < 0.05$ compared to the vehicle control of ectopic RUNX2-expressing cells. Data (DCF or MitoSox Red fluorescence relative to Calcein green fluorescence) are presented as Fold.

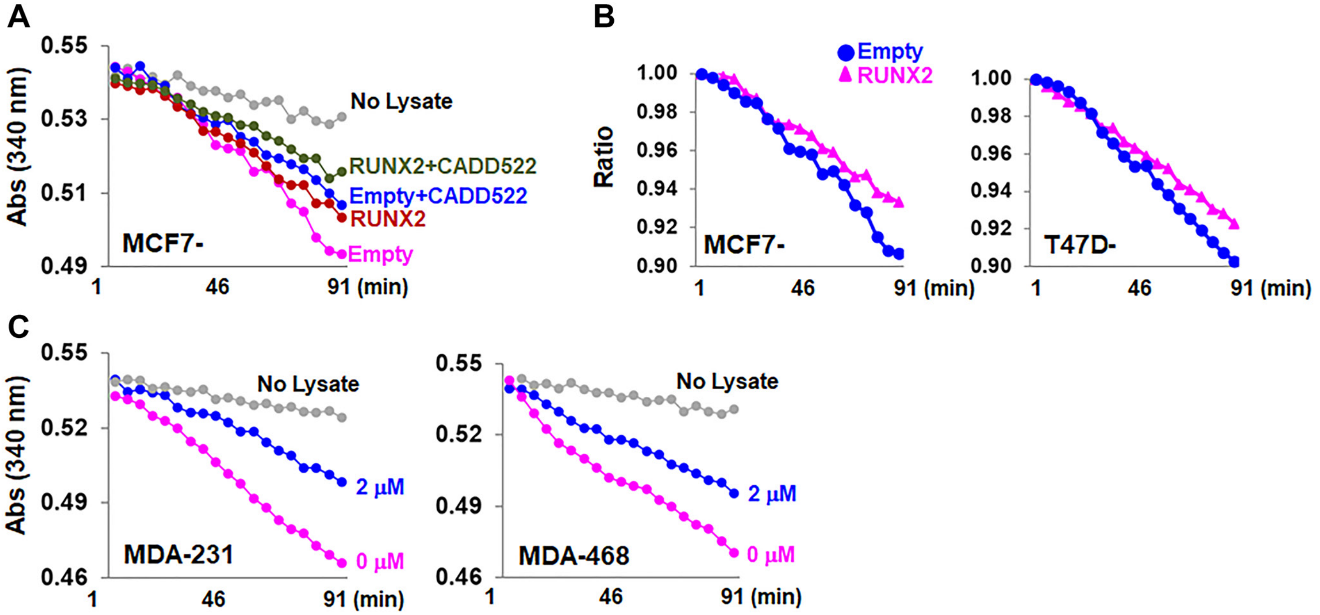


**Supplementary Figure 6:** (**A**) Cells were treated with CADD522 (50 μM) or vehicle (0.1% DMSO) alone for 24 hr. Data for the ATP synthase activity are presented as changes in absorbance at 340 nm. (**B**) Mitochondrial ATP synthase activity was compared in ectopic RUNX2 expressing MCF7 and T47D cells and their Empty cells. Data are presented as changes in relative value to the absorbance at 0 min (Ratio). (**C**) Cell lysates (50 μg) isolated from MDA-231 and MDA-468 cells that were not treated with CADD522 were directly incubated with CADD522 for 30 min, and the *in vitro* ATP synthase activity was determined. Data for the ATP synthase activity are presented as changes in absorbance at 340 nm.

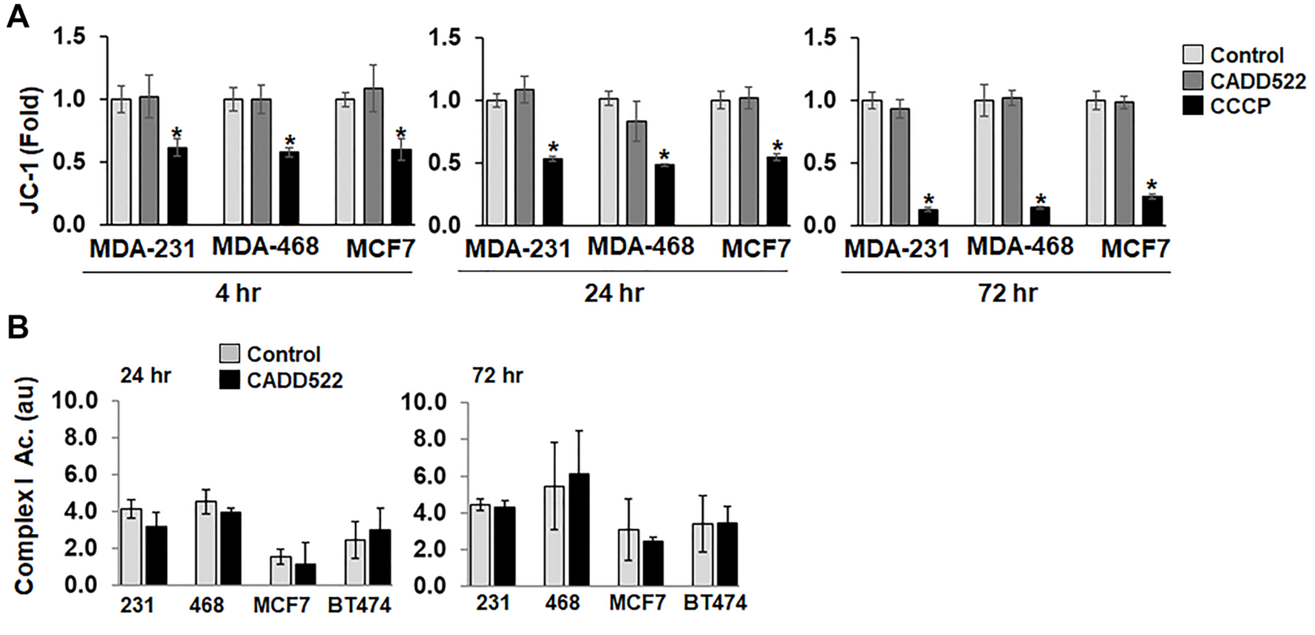


**Supplementary Figure 7:** (**A**) Cells were treated with CADD522 for 4~72 hr in complete growth medium and then incubated with JC-1. MMP (ΔΨm) was calculated as the ratio of the red to green fluorescence intensity of JC-1. Data are presented as the relative value to the vehicle control (Fold). Experiments were performed in four replicates and repeated twice (mean ± SD). CCCP (50 μM), a mitochondrial uncoupler, was used as a positive control for MMP reduction. CCCP treatment was for 30 min (left & middle) or 60 min (right) before incubating cells with JC-1. $^{*}P < 0.05$ compared to the vehicle control. (**B**) Complex I activity was determined using a commercial kit (Abcam) according to manufacturer's instruction. The activity level was indicated by the decrease in absorbance at 340 nm in kinetic mode, and data (mean ± SD) are presented in arbitrary units (au).

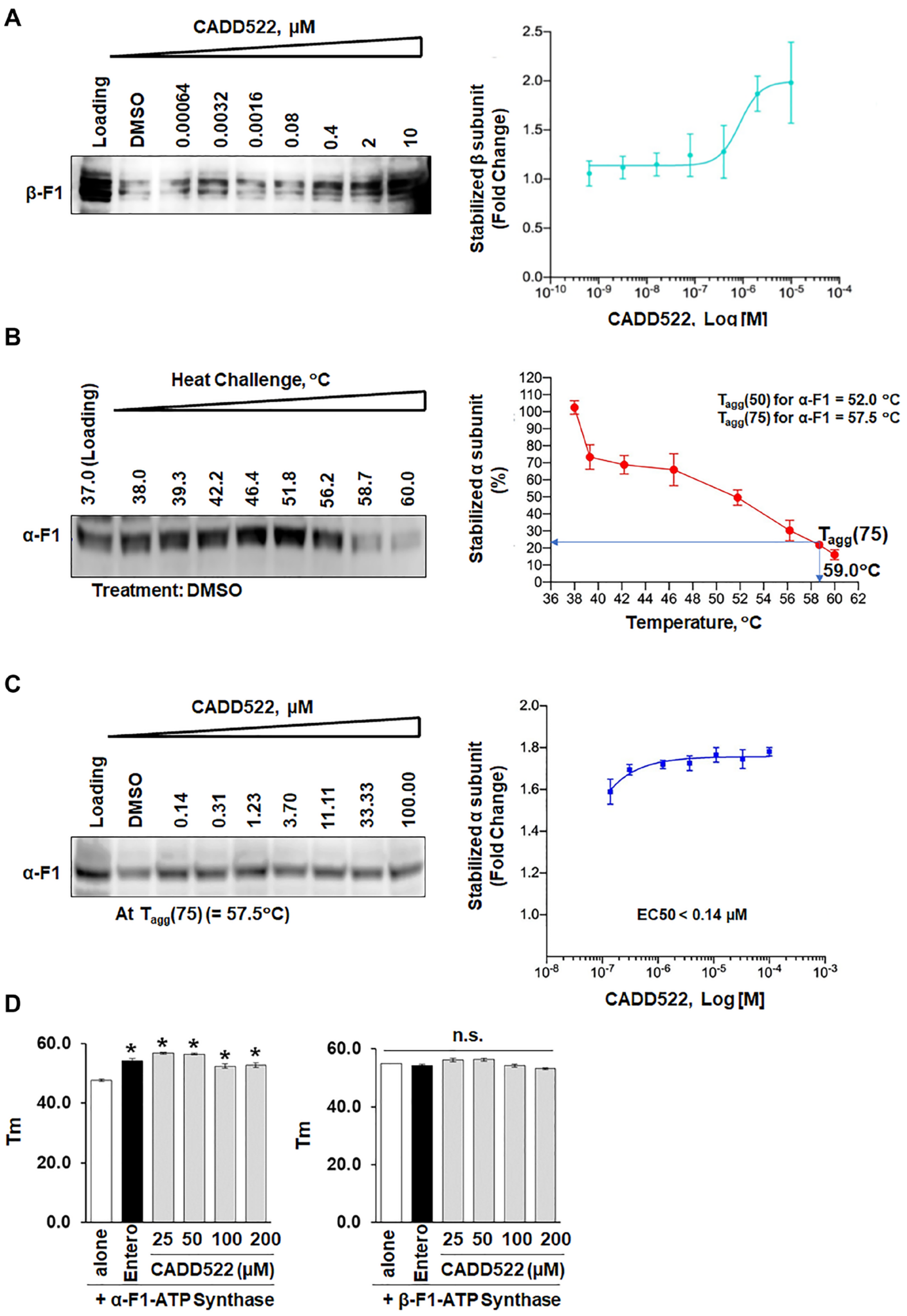


**Supplementary Figure 8:** (**A**) Dose-response target engagement of CADD522 at relatively low concentrations (≤10 µM) with F1-ATP synthase, β subunit. Experiments were performed in three replicates (mean ± SD) and repeated twice. β-F1, β-F1-ATP synthase. (**B**) Left: representative immunoblot result of the α-F1-ATP synthase upon heat challenges. Melting profile of α-F1-ATP synthase was first determined without CADD522 treatment. DMSO, vehicle control (0.1%); α-F1, α-F1-ATP synthase. Right, Thermal melting profile of the α-F1-ATP synthase. Band density of the the α subunit was quantified, normalized to loading (37°C), and analyzed for CETSA melting curves. Experiments were performed in three replicates (mean ± SD) and repeated twice. (**C**) Left: representative immunoblot result of the α-F1-ATP synthase upon increasing concentrations of CADD522. Right: dose-response target engagement of CADD522 in the α-F1-ATP synthase was performed at 57.5°C. Density of the the α subunit was quantified, normalized to the vehicle control (0.1% DMSO), and analyzed for melting curves. (**D**) DSF with recombinant α- or β-F1-ATP synthase. SYPRO orange, a fluorescent dye that binds to unfolded protein, 1.5 µM recombinant proteins, and CADD522 up to 200 µM were added to 96-well PCR plates, and melting curve analysis was performed. Experiments were performed in three replicates (mean ± SD) and repeated twice. $^{*}P < 0.05$ considered significant. Tm, the corresponding temperature of the midpoint from the fluorescence intensity. alone, α- or β-F1-ATP synthase only; Entero, Enterostatin (100 µM) with α- or β-F1-ATP synthase; n.s., not significant. Note, Enterostatin was reported to bind to purified β-F1-ATP synthase, which was determined by Surface Plasmon Resonance (SPR) measurement [1]. Results from DSF of this study show that enterostatin interacted with recombinant α-F1-ATP synthase rather than β-F1-ATP synthase.

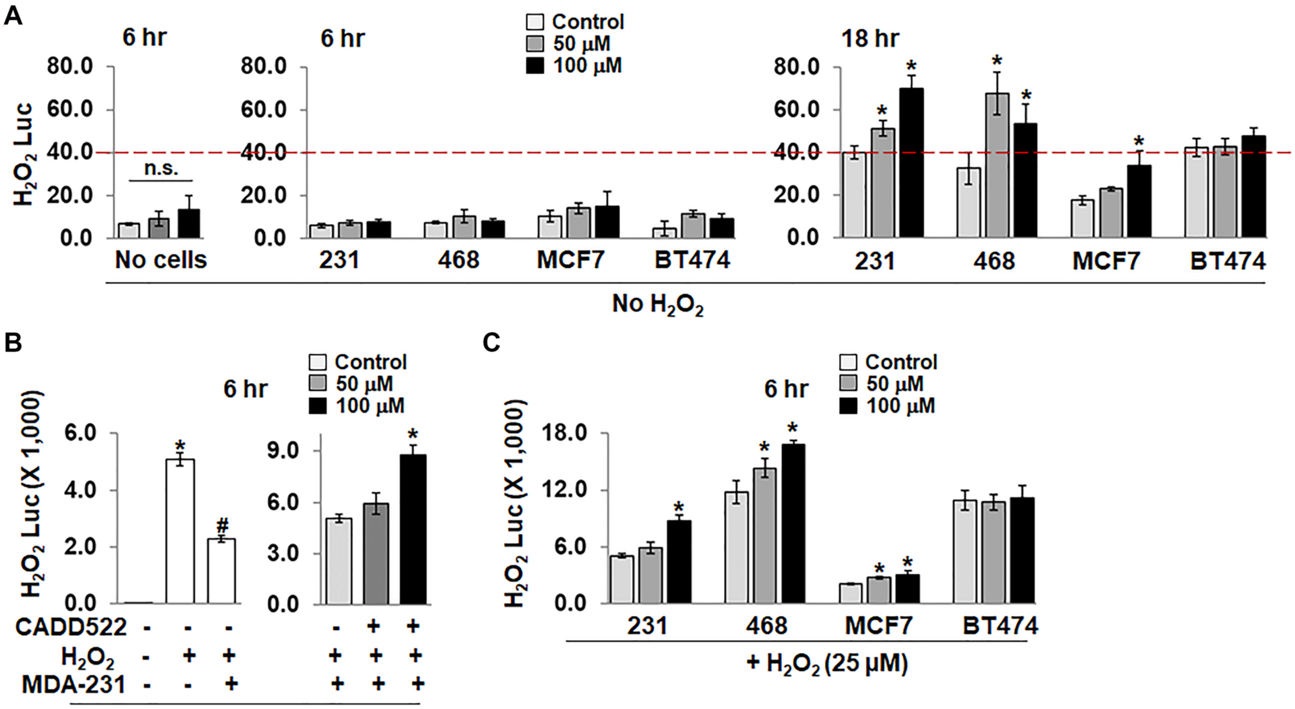


**Supplementary Figure 9:** (**A**) CADD522 (50 μM, 100 μM) was added in Complete M without exogenous $H_2O_2$, and incubated for 6 or 18 hr. The $H_2O_2$ Luc intensity was determined as described in Materials and Methods. Experiments were performed in triplicate and repeated twice (mean ± SD). $^*P < 0.05$ compared to Control. Red line, the intensity 50. (**B**) MDA-231 cells were cotreated with CADD522 and/or $H_2O_2$ (25 μM) for 6 hr, and the $H_2O_2$ Luc intensity was determined. Left, $^*P < 0.05$ compared to no treatment (first bar); $^\#P < 0.05$ compared to only $H_2O_2$ -treated samples (second bar). Right, $^*P < 0.05$ compared to Control. Note, The $H_2O_2$ Luc intensity indicates Values at Y-axis X 1,000. (**C**) Cells in Complete M were treated with CADD522 (0, 50, 100 μM) in the presence of $H_2O_2$ (25 μM) for 6 hr.

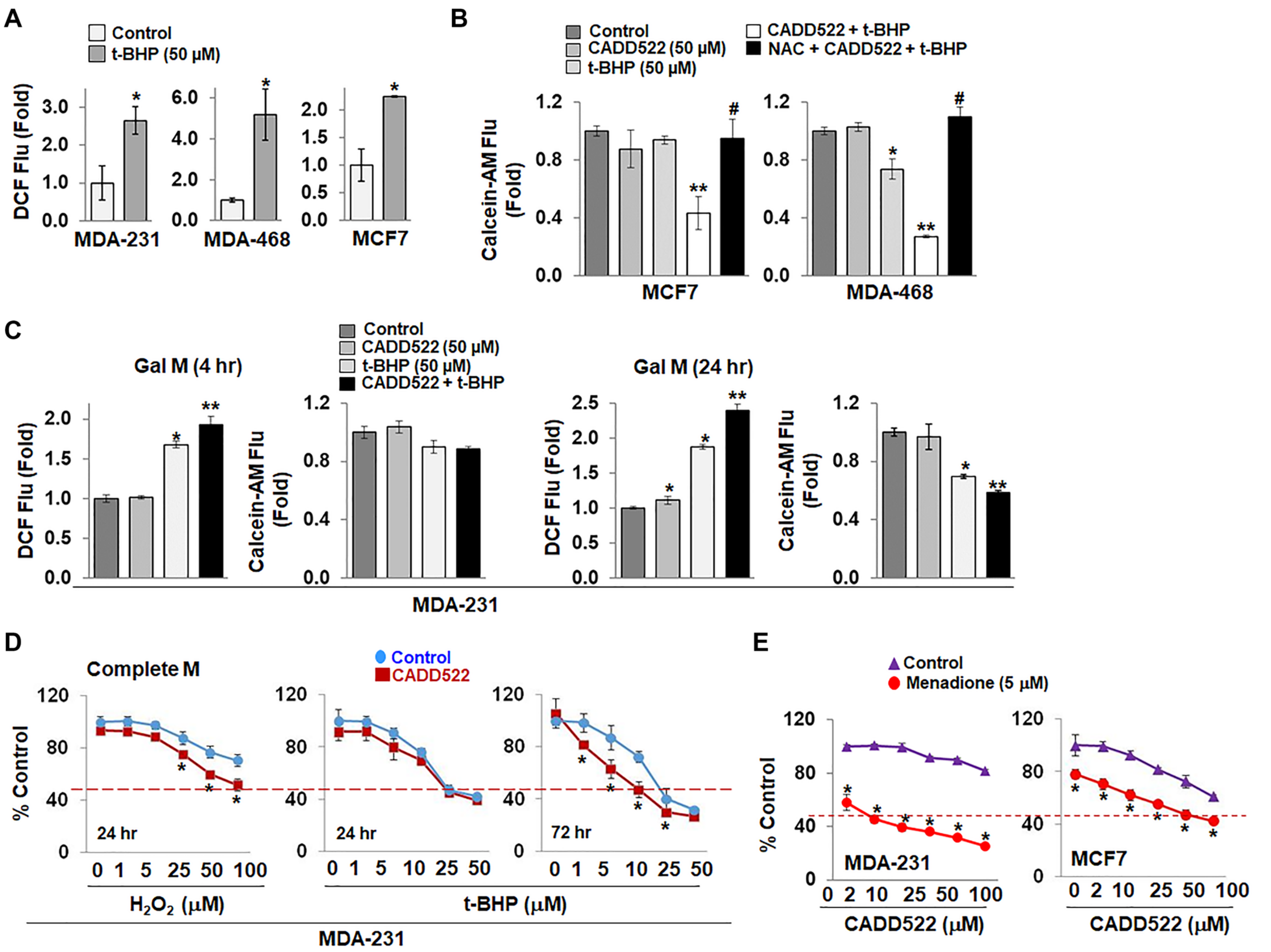


**Supplementary Figure 10:** (**A**) BC cells were treated with t-BHP for 4 hr in Complete M and intracellular ROS levels were determined with CM-H2DCFDA dye. Data (DCF fluorescence relative to Calcein green fluorescence) are presented as Fold, which was calculated from the relative value to the average of the vehicle control (0.1% DMSO, = 1). All determinations were in four replicates and repeated twice (mean ± SD). $^{*}P < 0.05$ compared to Control. (**B**) Cell viability was determined by Calcein-AM assay. NAC (5 mM) was pretreated for 1 hr and further treated with CADD522 and t-BHP for 4 hr in SFM. Data are presented as the relative value to Control (Fold). Experiments were done in four replicates and repeated twice (mean ± SD). $^{*}P < 0.05$ compared to Control; $^{**}P < 0.001$ compared to t-BHP alone; $^{\#}P < 0.001$ compared to CADD522 + t-BHP. (**C**) MDA-231 cells were treated with CADD522 (50 µM) and/or t-BHP for 4 hr or 24 hr in Galactose M, and intracellular ROS was determined with CM-H2DCFDA dye. Cell viability was determined by Calcein-AM assay, and data are presented as the relative value to Control (Fold). Experiments were performed in four replicates and repeated twice (mean ± SD). $^{*}P < 0.05$ compared to Control; $^{**}P < 0.001$ compared to t-BHP alone. (**D**) MDA-231 cells were treated with increasing concentrations of $H_2O_2$ or t-BHP in the absence or presence of CADD522 for 24~72 hr, and cell growth was determined by crystal violet staining. Data are presented as % Control. Red line, 50% of cell growth. (**E**) MDA-231 and MCF7 cell were treated concomitantly with Menadione (5 µM) and CADD522 for 72 hr and cell growth was determined by crystal violet staining. $^{*}P < 0.05$ compared to Control at indicated menadione concentrations.

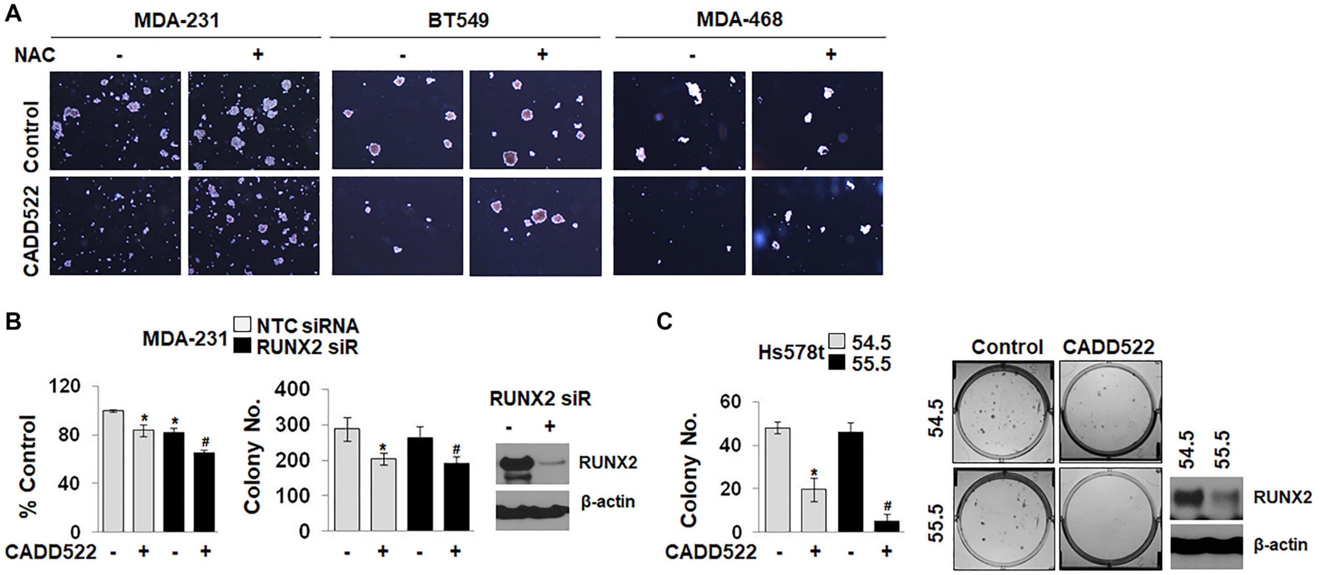


**Supplementary Figure 11:** (**A**) Tumorspheres were photographed at day 7 at ×40 magnifications and counted from 9 fields per well (described in Materials and Methods) (Sphere No.). (**B**) MDA-231 cells were transfected with RUNX2 siRNA and non-targeting control (NTC). After 24 hr, cells were trypsinized, counted and plated on 12-well plate and incubated overnight. Cells were treated with CADD522 (50 μM) for 72 hr and cell growth (left) was determined by crystal violet staining. For colony formation analysis, cells were plated on 6-well plate and treated with CADD522 for 9 days (middle). Data are presented as % control of NTC transfected cells (mean ± SD). Experiments were performed in triplicate and repeated twice. $^{*}P < 0.05$ compared to NTC was considered significant. $^{\#}P < 0.05$ compared to RUNX2-siRNA transfected cells with vehicle alone. In parallel, RUNX2 expression levels were determined by western blot analysis 48 hr after transfection (right). β-actin was used a loading control. (**C**) Colony forming ability of Hs578t cells with RUNX2 KD cells (55.5) and a negative clone of RUNX2 KD (54.5) were determined after cells were treated with CADD522 for 9 days. After crystal violet staining, colonies were taken picture of (middle) and counted (left). RUNX2 expression levels were determined by Western blot analysis.

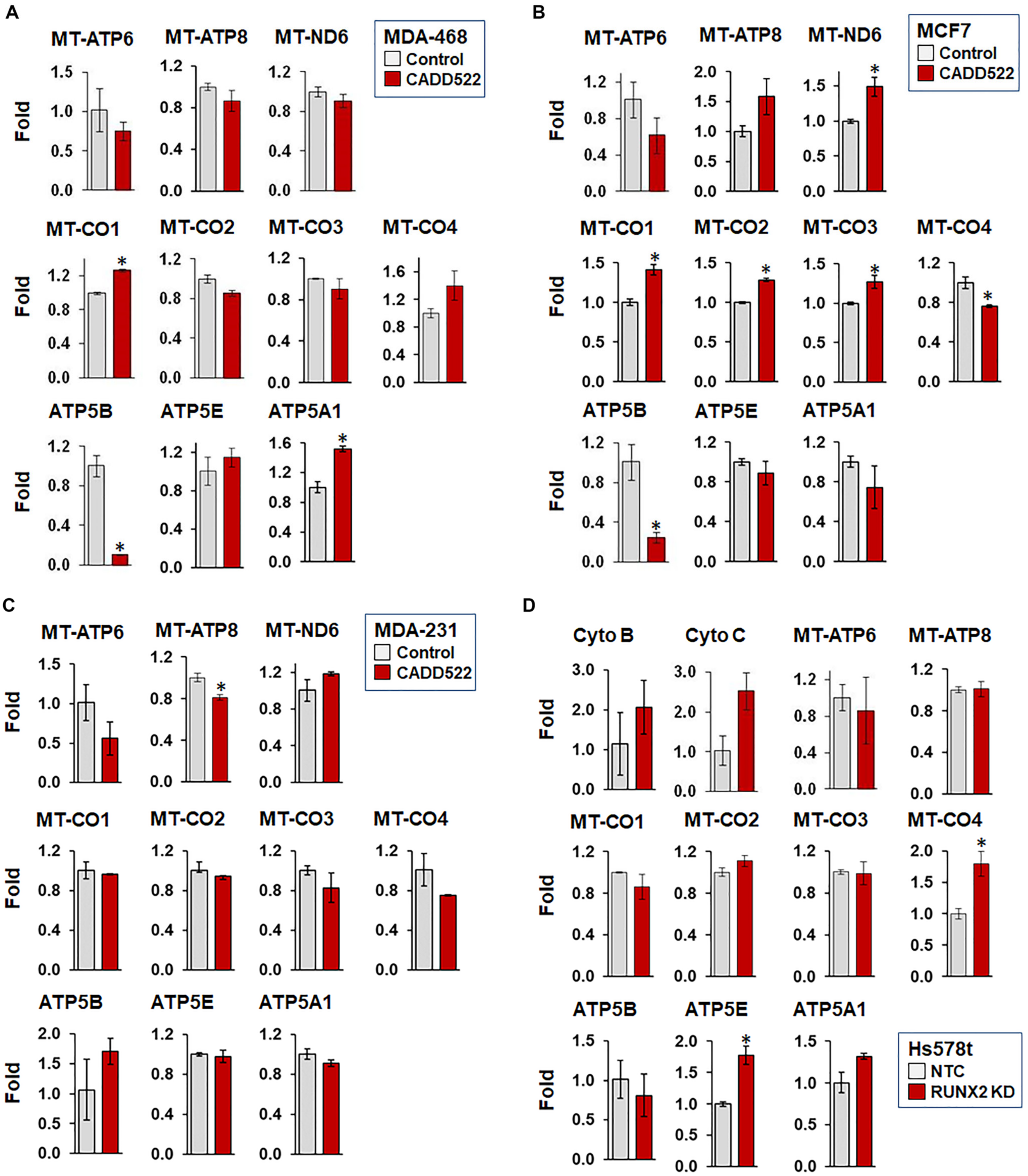
A
MT-ATP6
MT-ATP8
MT-ND6
MDA-468
Control
CADD522
MT-CO1
MT-CO2
MT-CO3
MT-CO4
ATP5B
ATP5E
ATP5A1
Fold
B
MT-ATP6
MT-ATP8
MT-ND6
MCF7
Control
CADD522
MT-CO1
MT-CO2
MT-CO3
MT-CO4
ATP5B
ATP5E
ATP5A1
Fold
C
MT-ATP6
MT-ATP8
MT-ND6
MDA-231
Control
CADD522
MT-CO1
MT-CO2
MT-CO3
MT-CO4
ATP5B
ATP5E
ATP5A1
Fold
D
Cyto B
Cyto C
MT-ATP6
MT-ATP8
MT-CO1
MT-CO2
MT-CO3
MT-CO4
ATP5B
ATP5E
ATP5A1
Hs578t
NTC
RUNX2 KD
Fold

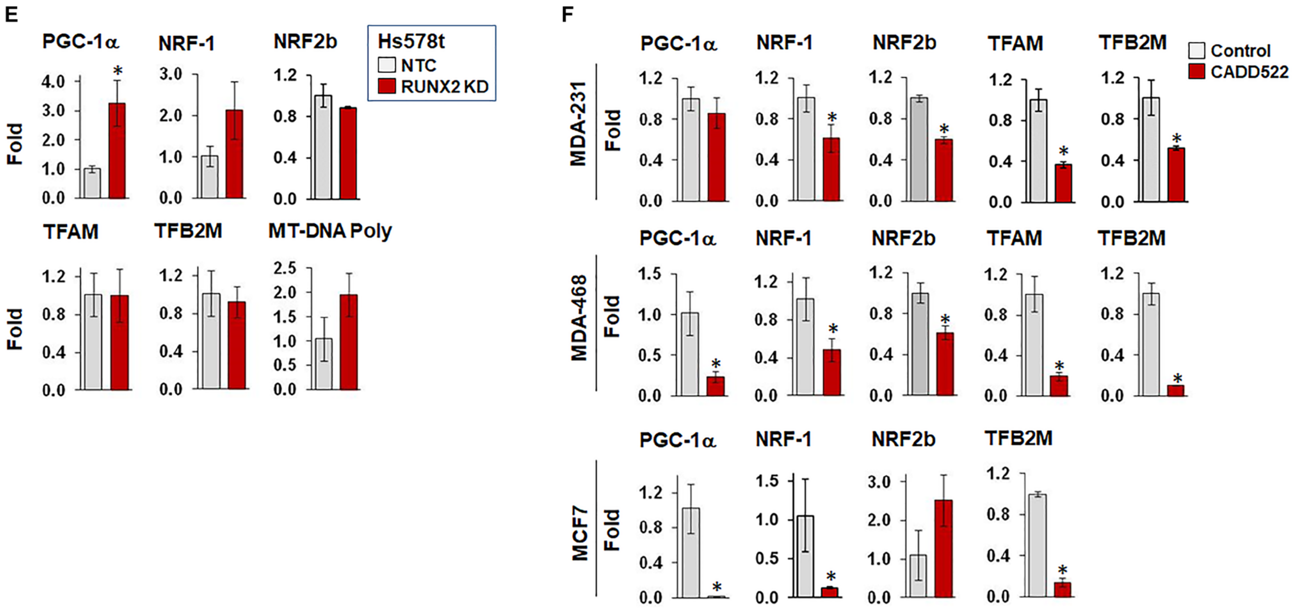


**Supplementary Figure 12: Changes in the mRNA level of mitochondria-related genes.** Q-RT-PCR analysis was performed in cDNA synthesized from total RNA of Hs578t cells with RUNX2 KD and the non-targeting control (NTC) (**A**, **E**) and BC cells treated with CADD522 (50 µM, 12 hr) or vehicle control (0.1% DMSO) (**B**, **C**, **D**, **F**). Gene expression levels relative to 18S rRNA level were normalized to vehicle control or NTC, and presented as Fold. Experiments were done in triplicate and repeated twice (mean ± SD). $^{*}P < 0.05$ compared to NTC or vehicle control.

## Supplementary Table 1: OCR response to CADD522

| Time (hrs) | Cell lines | CADD522 | OCR (pMoles/min/µg protein) | | | | % Inhibition {(A−B)/A × 100} | | | |
|---|---|---|---|---|---|---|---|---|---|---|
| | | | MRC | RC | AP | PL | MRC | RC | AP | PL |
| 6 | T47D-E | − | 60.83 | 15.11 | | | | | | |
| | | + | 46.05 | 8.17 | | | 24.29 | 45.94 | | |
| 6 | T47D-R | − | 71.77 | 28.81 | | | | | | |
| | | + | 52.53 | 18.22 | | | 26.81 | 36.76 | | |
| 24 | T47D-E | − | 129.30 | 66.40 | | | | | | |
| | | + | 121.89 | 53.94 | | | 5.73 | 18.76 | | |
| 24 | T47D-R | − | 127.77 | 66.36 | | | | | | |
| | | + | 82.77 | 31.17 | | | 35.22 | 53.03 | | |
| 72 | T47D-E | − | 36.13 | 15.97 | 9.65 | 10.51 | | | | |
| | | + | 27.96 | 11.65 | 6.64 | 9.68 | 22.61 | 27.06 | 31.26 | 7.89 |
| 72 | T47D-R | − | 24.28 | 10.06 | 7.05 | 7.17 | | | | |
| | | + | 20.70 | 9.61 | 2.37 | 8.72 | 14.75 | 4.45 | 66.44 | −21.61 |
| 6 | MCF7-E | − | 139.41 | 70.36 | | | | | | |
| | | + | 113.40 | 50.37 | | | 18.66 | 28.41 | | |
| 6 | MCF7-R | − | 121.40 | 57.51 | | | | | | |
| | | + | 88.26 | 36.62 | | | 27.30 | 36.32 | | |
| 30 | MCF7-E | − | 27.14 | 8.12 | 12.47 | 6.55 | | | | |
| | | + | 20.48 | 7.95 | 7.10 | 5.43 | 24.55 | 2.11 | 43.05 | 17.19 |
| 30 | MCF7-R | − | 29.17 | 12.79 | 10.63 | 5.75 | | | | |
| | | + | 11.00 | 2.15 | 7.50 | 1.35 | 62.30 | 83.19 | 29.49 | 76.49 |
| 72 | MCF7-E | − | 88.08 | 41.42 | 25.23 | 21.42 | | | | |
| | | + | 55.70 | 20.40 | 43.90 | −8.60 | 36.76 | 50.75 | −73.98 | 140.15 |
| 72 | MCF7-R | − | 76.90 | 32.53 | 33.14 | 11.23 | | | | |
| | | + | 37.15 | −1.07 | 21.39 | 16.83 | 51.69 | 103.28 | 35.46 | −49.90 |
| 6 | MDA-468 | − | 113.16 | 61.05 | | | | | | |
| | | + | 90.53 | 51.16 | | | 20.00 | 16.19 | | |
| 24 | MDA-468 | − | 110.22 | 62.80 | | | | | | |
| | | + | 42.99 | 16.44 | | | 61.00 | 73.83 | | |
| 24 | MDA-468 | − | 96.61 | 93.48 | 14.67 | 39.41 | | | | |
| | | + | 38.30 | 56.60 | 6.91 | 17.85 | 60.36 | 39.46 | 52.90 | 54.70 |
| 6 | MCF7 | − | 47.26 | 3.86 | | 36.16 | | | | |
| | | + | 27.43 | 0.59 | | 26.32 | 41.96 | 84.61 | | 27.21 |

Data shown as mean values of OCR and % inhibition presented in Figure 1 and Supplementary Figure 1. Cells were treated with CADD522 (50 µM) for 6~72 hrs. −, vehicle control; +, CADD522 treatment. % Inhibition was calculated from the equation (A−B)/A × 100. A, cells with vehicle control; B, cells with CADD522 treatment. % Inhibition of the individual parameters of OCR; MRC, Maximal Respiration Capacity; RC, Reserve Capacity; AP, ATP production-linked OCR; PL, Proton leak-linked OCR; BL, Baseline OCR; NM, non-mitochondrial OCR.

## Supplementary Table 2: ATP synthase activity

| | | mean ± SD | $^{a}P$ | $^{b}P$ |
|---|---|---|---|---|
| Cells + CADD522 (24 hrs) | | | | |
| | CADD522 (50 µM) | | | |
| MCF7-Empty | - | 0.53 ± 0.05 | | |
| | + | 0.37 ± 0.03 | *0.012* | |
| | | | | *0.029* |
| MCF7-RUNX2 | - | 0.42 ± 0.02 | | |
| | + | 0.30 ± 0.02 | *0.005* | |
| Lysates + CADD522 (30 min) | | | | |
| | CADD522 (nM) | | | |
| MDA-231 | 0 | 1.36 ± 0.14 | | |
| | 50 | 1.18 ± 0.18 | *n/s* | |
| | 250 | 1.18 ± 0.15 | *n/s* | |
| | 2000 | 0.85 ± 0.05 | *0.004* | |
| MDA-468 | 0 | 1.43 ± 0.12 | | |
| | 500 | 1.14 ± 0.03 | *0.017* | |
| | 2000 | 1.05 ± 0.20 | *0.001* | |

$^{a}P$ values from CADD522-treated cells vs. vehicle control ($n = 3$). $^{b}P$ value from MCF7-Empty vs. MCF7-RUNX2 cells. n/s, not significant ($P > 0.05$).

## Supplementary Table 3: Sensitivity to MDA-231 cells (%)

| | CADD522 | | |
|---|---|---|---|
| | 0 µM | 10 µM | 50 µM |
| Vehicle | 100.00 ± 3.26 | 83.69 ± 1.69 | 79.35 ± 1.95 |
| 2-DG (2 mM) | 72.26 ± 3.33 | 72.59 ± 1.50 | $^{*}$49.45 ± 3.47 |
| 3-BPA (25 µM) | 93.92 ± 1.52 | 93.32 ± 3.40 | 87.15 ± 1.81 |
| DCA (10 mM) | 95.12 ± 11.42 | 96.08 ± 4.66 | 71.37 ± 1.80 |
| IAA (5 µM) | 103.02 ± 1.35 | 89.19 ± 12.58 | 75.20 ± 6.83 |

Sensitivity was determined by crystal violet staining assay. CADD522 with or without inhibitors were treated for 72 hrs. Vehicle, 0.1% DMSO. $^{*}P < 0.05$ in comparison between CADD522 with and without inhibitors. 2-DG, 2-deoxyglucose; 3-BPA, 3-bromopyruvic acid; DCA, dichloroacetate; IAA, iodoacetic acid.

## Supplementary Table 4: Primers for Q-RT-PCR analysis

| Gene | FW (5′–3′) | RV (5′–3′) | |
|---|---|---|---|
| MT-ATP6 | TCGCTTTGTAACCCTCCAAC | GGGATGGCTATGCCTAGGTT | ATP synthase Fo subunit 6 |
| MT-ATP8 | CCCCCATACTCCTTACACTATTCC | GGGCTTTGGTGAGGGAGGT | ATP Synthase Fo Subunit 8 |
| MT-ND6 | TAATCATACAAAGCCCCCGC | TAGG ATTGGTGCTGTGGGTG | NADH-Ubiquinone Oxidoreductase Chain 6 |
| MT-CO1 | CTATACCTATTATTCGGCGCATGA | CAGCTCGGCTCGAATAAGGA | Cytochrome C Oxidase Subunit 1 |
| MT-CO2 | CTGAACCTACGAGTACACCG | TTAATTCTAGGACGATGGGC | Cytochrome C Oxidase Subunit 2 |
| MT-CO3 | CGGCCTAGCCATGTGATTTC | CGCGCCATCATTGGTATATG | Cytochrome C Oxidase Subunit 3 |
| MT-CO4 | GCCATGTTCTTCATCGGTTTC | GGCCGTACACATAGTGCTTCTG | Cytochrome C Oxidase Subunit 4 |
| ATP5B | ACAGGACCCTATGTGCTTGG | ATCAGCAAATTCCCCAACAG | The β subunit of the F1 ATP Synthase |
| ATP5A1 | CTGGGAAAACCTCAATTGCT | GATCTCTTTTGACCAATAGCAACA | The α subunit of the F1 ATP Synthase |
| ATP5E | CCGCTTCTGTGGTCTGATCT | CAGTAGGCCACCATGCTGTA | The ε subunit of the F1 ATP Synthase |
| MT-CYB | AGACAGTCCCACCCTCACAC | GTTGTTTGATCCCGTTTCGT | Cytochrome b |
| MT-CYC | CCAGGTAGCCAAGGATGTGT | GACCCTGAAGCTCAGGACAG | Cytochrome C |
| PGC-1α | TCATGCCGTGGTAAGTACCA | GTGCAAAGTTCCCTCTCTGC | Perroxisome proliferator-activated receptor γ coactivator 1-α |
| NRF1 | ACCGCCGAATAATTCACTTG | CACAAACACAGGCCACAACC | Nuclear respiratory factor 1 |
| NRF2b | CATTGTGACCATGCCAGATG | GTAGGCCTCTGCTTCCTGTTC | NRF2b/GABP-b |
| TFAM | GGGAGGAACAAATGATGGAA | CCATGGGCTACAGAAAAGGA | Mitochondrial transcription factor A |
| TFB2M | GTACAAGTCCCGTTCCGAGAC | CACTCTGGCACCACTTTCAAG | Mitochondrial transcription factor B2 |
| 18S rRNA | GAGGATGAGGTGGAACGTGT | AGAAGTGACGCAGCCCTCTA | 18S ribosomal RNA |

## SUPPLEMENTARY REFERENCES


1. Park M, Lin L, Thomas S, Braymer HD, Smith PM, Harrison DH, York DA. The F1-ATPase beta-subunit is the putative enterostatin receptor. Peptides. 2004; 25:2127–33. https://doi.org/10.1016/j.peptides.2004.08.022. [PubMed]
